# Association of High Tumor Mutation Burden in Non–Small Cell Lung Cancers With Increased Immune Infiltration and Improved Clinical Outcomes of PD-L1 Blockade Across PD-L1 Expression Levels

**DOI:** 10.1001/jamaoncol.2022.1981

**Published:** 2022-06-16

**Authors:** Biagio Ricciuti, Xinan Wang, Joao V. Alessi, Hira Rizvi, Navin R. Mahadevan, Yvonne Y. Li, Andrew Polio, James Lindsay, Renato Umeton, Rileen Sinha, Natalie I. Vokes, Gonzalo Recondo, Giuseppe Lamberti, Marissa Lawrence, Victor R. Vaz, Giulia C. Leonardi, Andrew J. Plodkowski, Hersh Gupta, Andrew D. Cherniack, Michael Y. Tolstorukov, Bijaya Sharma, Kristen D. Felt, Justin F. Gainor, Arvind Ravi, Gad Getz, Kurt A. Schalper, Brian Henick, Patrick Forde, Valsamo Anagnostou, Pasi A. Jänne, Eliezer M. Van Allen, Mizuki Nishino, Lynette M. Sholl, David C. Christiani, Xihong Lin, Scott J. Rodig, Matthew D. Hellmann, Mark M. Awad

**Affiliations:** 1 Lowe Center for Thoracic Oncology, Dana-Farber Cancer Institute, Harvard Medical School, Boston, Massachusetts; 2 Department of Environmental Health, Harvard T.H. Chan School of Public Health, Harvard University, Boston, Massachusetts; 3 Department of Medicine, Weill Cornell Medical College, Memorial Sloan Kettering Cancer Center, New York, New York; 4 Department of Pathology, Brigham and Women’s Hospital, Boston, Massachusetts; 5 Department of Medical Oncology, Dana-Farber Cancer Institute, Harvard Medical School, Boston, Massachusetts; 6 Cancer Program, Broad Institute of MIT and Harvard, Cambridge, Massachusetts; 7 Knowledge Systems Group, Dana-Farber Cancer Institute, Boston, Massachusetts; 8 Department of Informatics and Analytics, Dana-Farber Cancer Institute, Boston, Massachusetts; 9 Department of Thoracic/Head and Neck Oncology, MD Anderson Cancer Center, Houston, Texas; 10 Department of Radiology, Memorial Sloan Kettering Cancer Center, New York, New York; 11 ImmunoProfile, Brigham and Women’s Hospital and Dana-Farber Cancer Institute, Boston, Massachusetts; 12 Department of Medicine, Massachusetts General Hospital Cancer Center, Boston; 13 Broad Institute of MIT and Harvard, Cambridge, Massachusetts; 14 Department of Pathology, Yale School of Medicine, New Haven, Connecticut; 15 Department of Medicine, Columbia University Medical Center, New York, New York; 16 The Sidney Kimmel Comprehensive Cancer Center, Johns Hopkins University School of Medicine, Baltimore, Maryland; 17 Belfer Center for Applied Cancer Science, Dana-Farber Cancer Institute, Boston, Massachusetts; 18 Center for Cancer Precision Medicine, Dana-Farber Cancer Institute, Boston, Massachusetts; 19 Department of Radiology, Brigham and Women’s Hospital, Boston, Massachusetts; 20 Department of Biostatistics, Harvard T.H. Chan School of Public Health, Harvard University, Boston, Massachusetts; 21 Center for Immuno-Oncology, Dana-Farber Cancer Institute, Boston, Massachusetts

## Abstract

**Importance:**

Although tumor mutation burden (TMB) has been explored as a potential biomarker of immunotherapy efficacy in solid tumors, there still is a lack of consensus about the optimal TMB threshold that best discriminates improved outcomes of immune checkpoint inhibitor therapy among patients with non–small cell lung cancer (NSCLC).

**Objectives:**

To determine the association between increasing TMB levels and immunotherapy efficacy across clinically relevant programmed death ligand–1 (PD-L1) levels in patients with NSCLC.

**Design, Setting, and Participants:**

This multicenter cohort study included patients with advanced NSCLC treated with immunotherapy who received programmed cell death–1 (PD-1) or PD-L1 inhibition in the Dana-Farber Cancer Institute (DFCI), Memorial Sloan Kettering Cancer Center (MSKCC), and in the Stand Up To Cancer (SU2C)/Mark Foundation data sets. Clinicopathological and genomic data were collected from patients between September 2013 and September 2020. Data analysis was performed from November 2021 to February 2022.

**Exposures:**

Treatment with PD-1/PD-L1 inhibition without chemotherapy.

**Main Outcomes and Measures:**

Association of TMB levels with objective response rate (ORR), progression-free survival (PFS), and overall survival (OS).

**Results:**

In the entire cohort of 1552 patients with advanced NSCLC who received PD-1/PD-L1 blockade, the median (range) age was 66 (22-92) years, 830 (53.5%) were women, and 1347 (86.8%) had cancer with nonsquamous histologic profile. A regression tree modeling ORR as a function of TMB identified 2 TMB groupings in the discovery cohort (MSKCC), defined as low TMB (≤19.0 mutations per megabase) and high TMB (>19.0 mutations per megabase), which were associated with increasing improvements in ORR, PFS, and OS in the discovery cohort and in 2 independent cohorts (DFCI and SU2C/Mark Foundation). These TMB levels also were associated with significant improvements in outcomes of immunotherapy in each PD-L1 tumor proportion score subgroup of less than 1%, 1% to 49%, and 50% or higher. The ORR to PD-1/PD-L1 inhibition was as high as 57% in patients with high TMB and PD-L1 expression 50% or higher and as low as 8.7% in patients with low TMB and PD-L1 expression less than 1%. Multiplexed immunofluorescence and transcriptomic profiling revealed that high TMB levels were associated with increased CD8-positive, PD-L1–positive T-cell infiltration, increased PD-L1 expression on tumor and immune cells, and upregulation of innate and adaptive immune response signatures.

**Conclusions and Relevance:**

These findings suggest that increasing TMB levels are associated with immune cell infiltration and an inflammatory T-cell–mediated response, resulting in increased sensitivity to PD-1/PD-L1 blockade in NSCLC across PD-L1 expression subgroups.

## Key Points

**Question:**

Is tumor mutation burden (TMB) associated with improved outcomes of programmed cell death–1 (PD-1)/programmed death ligand–1 (PD-L1) inhibition across PD-L1 expression levels in non–small cell lung cancer (NSCLC)?

**Findings:**

In this cohort study of 1552 patients with NSCLC, the group with high TMB had improved response rates and survival after receiving PD-1/PD-L1 inhibition therapy across PD-L1 expression subgroups compared with the group with low TMB. High TMB levels were associated with increased CD8-positive T-cell infiltration and distinct immune response gene expression signatures.

**Meaning:**

These findings suggest that in NSCLC, a high number of nonsynonymous tumor mutations is associated with immune cell infiltration and inflammatory T-cell expression signatures, leading to increased sensitivity to PD-1/PD-L1 inhibition across PD-L1 expression subgroups.

## Teaser

This cohort study examines the association between increasing tumor mutation burden (TMB) levels and immunotherapy efficacy across clinically relevant programmed death ligand–1 (PD-L1) levels in patients with non–small cell lung cancer (NSCLC).

## Introduction

Immune checkpoint inhibitors (ICIs) are an integral component of standard treatment for the majority of patients with advanced non–small cell lung cancer (NSCLC).^1,2,3,4^ However, the degree of benefit associated with ICI therapy is highly variable, and the identification of clinically available biomarkers of response to ICIs in NSCLC has been challenging. Although programmed death ligand–1 (PD-L1) expression levels are associated with response to immunotherapy in NSCLC,^2,5^ lung cancers across all PD-L1 expression levels may respond to ICIs. In addition, PD-L1 expression is temporally and spatially heterogeneous,^6^ further highlighting the need for identifying additional precise biomarkers of immunotherapy efficacy. Tumor mutation burden (TMB), defined as the total number of nonsynonymous mutations per sequenced coding area of a tumor genome, has emerged as a potential factor associated with ICI efficacy across different tumor types.^7^ However, in NSCLC, despite several large prospective clinical trials aimed at establishing TMB as a robust biomarker of ICI therapy,^8,9,10^ they have not consistently demonstrated an overall survival benefit; therefore, the role of TMB as a biomarker in NSCLC remains elusive. Here, we analyzed multiple independent cohorts of patients with NSCLC treated with programmed cell death–1 (PD-1)/PD-L1 inhibitors to identify clinicopathological, genomic, and immunophenotypic correlates of TMB, and to investigate TMB groupings that best discriminate responders from nonresponders to ICIs.

## Methods

This study was approved by the Dana-Farber Cancer Institute institutional review board. All patients provided written informed consent at enrollment in the respective cohorts. This cohort study follows the Strengthening the Reporting of Observational Studies in Epidemiology (STROBE) reporting guideline. Patients from the Memorial Sloan Kettering Cancer Center (MSKCC), Dana-Farber Cancer Institute (DFCI), and the Stand Up To Cancer (SU2C)/Mark Foundation data set whose tumors underwent genomic profiling with MSK-IMPACT, DFCI-OncoPanel, or whole-exome sequencing, respectively, were included.

### Statistical Analysis

The TMB distributions were normalized across different platforms by applying a normal transformation followed by standardization to *z* scores, as described elsewhere.^11^ An unbiased regression tree^12^ was used to identify the optimal TMB cutoff regarding objective response in the MSKCC discovery cohort, and this cutoff was then externally validated in the DFCI and SU2C/Mark Foundation cohorts in both univariable and multivariable Cox regression analyses (eFigure 1 in the Supplement). Significance was set at 2-sided *P* < .05. The TMB comparisons were computed using the Mann-Whitney *U *test or the Kruskal-Wallis test, when appropriate. Linear correlations were evaluated using Spearman test, and categorical variables were evaluated using Fisher exact test. All statistical analyses were performed using R statistical analysis version 3.6.3 (R Project for Statistical Computing). Data analysis was performed from November 2021 to February 2022. Detailed methods, including statistical analysis, methods used for TMB assessment, genomic, transcriptomic, and immunophenotypic analysis, are reported in the eAppendix in the Supplement.

## Results

### Patient Characteristics

A total of 3591 NSCLC samples at DFCI that underwent tumor genomic profiling were used to identify clinical, histologic, and genomic characteristics associated with TMB, summarized in eTable 1 in the Supplement. The median (range) age was 66 (18-99) years, and 78.3% of patients had a history of tobacco use. In the entire cohort of 1552 patients with advanced NSCLC who received PD-L1 blockade, the median (range) age was 66 (22-92) years, 830 (53.5%) were women, and 1347 (86.8%) had cancer with nonsquamous histologic profile. The median (range) TMB was 9.8 (0-104.9) mutations per megabase (eFigure 2A in the Supplement). The TMB values were highest among current smokers, followed by former smokers, and lowest among never smokers (eFigure 2B in the Supplement); there was a linear association between TMB and pack-years of tobacco use (eFigure 2C in the Supplement). The TMB distributions were comparable in squamous and nonsquamous histologic profiles among tobacco-associated NSCLCs (eFigure 2D in the Supplement). The TMB was higher in patients with stage II, III, and IV NSCLCs compared with those with stage I NSCLCs (eFigure 2E in the Supplement). When analyzed by oncogenic mutation status, NSCLCs with activating mutations in *BRAF* and *KRAS* had the highest TMB, as did those without an identifiable driver mutation, whereas NSCLCs with *EGFR* mutations and chromosomal rearrangements in *RET* and *ALK* had the lowest TMB of the cases examined (eFigure 3A and 3B in the Supplement).

### Association of TMB With Clinical Outcomes of PD-1/PD-L1 Inhibition in NSCLC

We next investigated the association of TMB with clinical outcomes among patients who received ICI at MSKCC (672 patients), DFCI (714 patients), and SU2C (166 patients) (eTable 2 in the Supplement). In each of the 3 cohorts, tumors from responders to immunotherapy had significantly higher TMB compared with those from patients with stable or progressive disease (*P* < .001, Kruskal-Wallis analysis of variance) (eFigure 4 in the Supplement), consistent with previous reports.^13,14^ Given inconsistent results from previous studies exploring the association of increasing TMB levels with ICI efficacy in different tumor types,^15,16,17,18^ we next sought to determine whether there was an optimal threshold of TMB that discriminated responders from nonresponders to ICI specifically in NSCLC, leveraging data from multiple centers using a statistically robust framework. Because TMB was estimated with different platforms in the MSKCC (MSK-IMPACT), DFCI (DFCI OncoPanel), and in the SU2C/Mark Foundation cohorts (whole-exome sequencing), we first harmonized the TMB distribution across the 3 platforms by applying a normal transformation followed by standardization to *z* scores, as described elsewhere^11^ (eFigure 5 in the Supplement). To identify an optimal TMB cutoff, we first fitted a regression tree in the MSKCC discovery cohort modeling the response to ICI as a function of normalized TMB. In this discovery cohort, a TMB *z* score of greater than 1.16, corresponding to 19.0 mutations per megabase on the MSK-IMPACT platform, identified patients with the greatest likelihood of responding to ICI (eFigure 6A in the Supplement). Patients with a TMB greater than 19.0 mutations per megabase had significantly higher objective response rate (ORR) to ICI (42.5% vs 18.0%; difference, 24.5%; 95% CI, 12.7%-36.2%; *P* < .001), and longer progression-free survival (PFS) (hazard ratio [HR], 0.38; 95% CI, 0.28-0.52; *P* < .001) and overall survival (OS) (HR, 0.46; 95% CI, 0.32-0.65; *P* < .001) compared with those with a TMB less than or equal to 19.0 mutations per megabase (eFigure 6B, 6C, and 6D in the Supplement). We next validated the impact of this normalized TMB *z* score cutoff of 1.16 in the DFCI and SU2C/Mark Foundation cohorts. In the DFCI cohort, we confirmed that patients with high-TMB NSCLC (>1.16 *z* score, which corresponded to >19.3 mutations per megabase in this cohort) had a significantly higher ORR to ICI (44.9% vs 21.1%; difference, 23.8%; 95% CI, 11.6%-35.9%; *P* < .001) (eFigure 7A in the Supplement) and significantly improved PFS (HR, 0.50; 95% CI, 0.37-0.67; *P* < .001) (eFigure 7B in the Supplement) and OS (HR, 0.55; 95% CI, 0.39-0.77; *P* < .001) (eFigure 7C in the Supplement) compared with patients with low TMB (≤19.3 mutations per megabase). Similarly, in the SU2C/Mark Foundation data set, patients with a high TMB *z* score (>1.16), corresponding to more than 16.0 mutations per megabase in this cohort, had significantly higher ORR (89.5% vs 37.4%; difference, 52.1%; 95% CI, 36.2%-67.9%; *P* < .001) (eFigure 7D in the Supplement) and longer PFS (HR, 0.18; 95% CI, 0.08-0.41; *P* < .001) (eFigure 7E in the Supplement) and OS (HR, 0.18; 95% CI, 0.06-0.57; *P* = .003) (eFigure 7F in the Supplement) compared with those with a TMB less than or equal to 16.0 mutations per megabase. The TMB thresholds corresponding to the normalized TMB *z* score of 1.16 based on different platforms across the 3 cohorts is shown in eTable 3 in the Supplement; this value corresponded to the approximately 90th percentile for TMB in each of the cohorts. Importantly, a high TMB retained a significant association with improved ORR, PFS, and OS in multivariable analysis in each of the 3 independent cohorts (eFigures 8-10 in the Supplement). Multivariable sensitivity analysis using inverse probability weights^19^ (eFigures 11-13 in the Supplement) and multiple imputation^20^ (eTables 4-6 in the Supplement) were conducted to account for potential selection bias resulting from PD-L1 missingness, and confirmed that a high TMB was an independent factor associated with improved ORR, PFS, and OS in each of the 3 independent cohorts.

**Figure 1.**
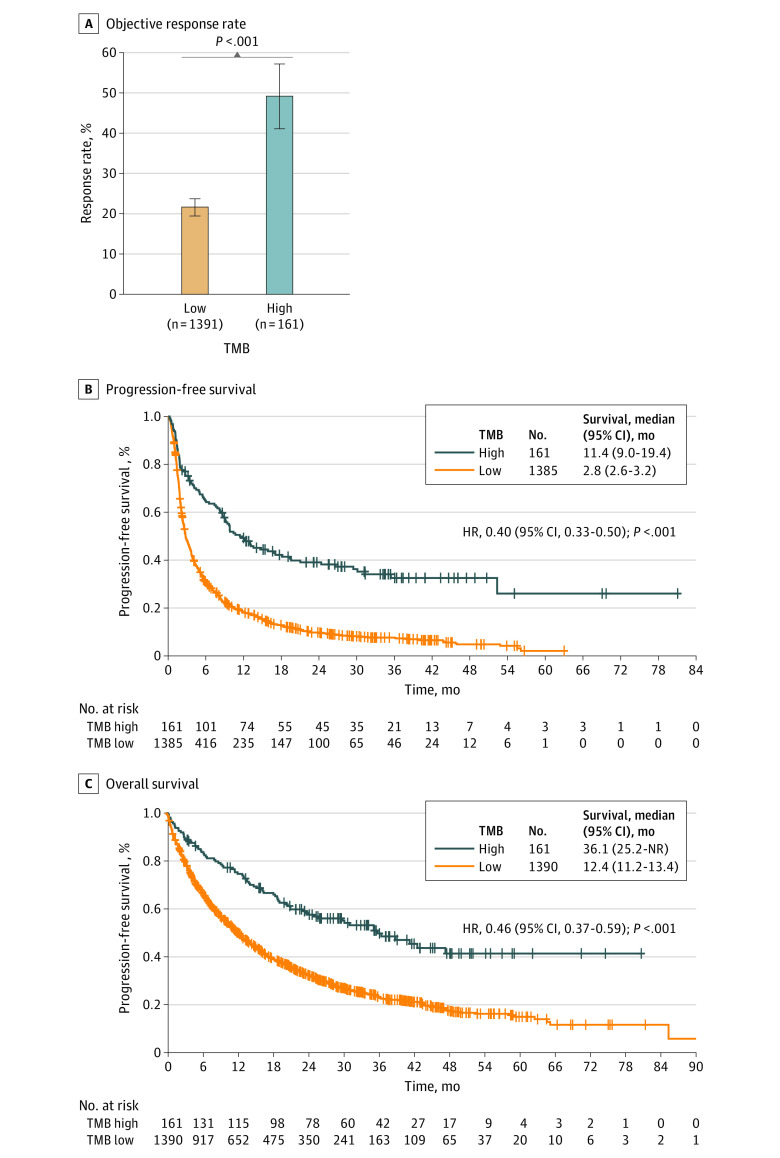
Objective Response Rate, Progression-Free Survival, and Overall Survival in Patients With a High vs Low Harmonized Tumor Mutation Burden (TMB) Data are from the pooled cohort of 1552 patients with non–small cell lung cancer treated with programmed death ligand–1 blockade from the Dana-Farber Cancer Institute, Memorial Sloan Kettering Cancer Center, and Stand Up To Cancer/Mark Foundation data sets. HR indicates hazard ratio; NR, not reached.

Having demonstrated that a high TMB was associated with improved clinical outcomes of immunotherapy in 3 independent cohorts of patients, we further evaluated the association of this TMB threshold in a pooled analysis of the MSKCC, DFCI, and SU2C/Mark Foundation cohorts. We confirmed that patients with NSCLC and a high harmonized TMB *z* score of 1.16 or higher (corresponding to ≥19.0 mutations per megabase for the MSKCC, ≥19.3 mutations per megabase for the DFCI cohort, and ≥16.0 mutations per megabase for the SU2C/Mark Foundation cohort) had significantly higher ORR (49.1% vs 21.5%; difference, 27.6%; 95% CI, 19.6%-35.6%; *P* < .001) and significantly longer PFS (11.4 vs 2.8 months; HR, 0.40; 95% CI, 0.33-0.50; *P* < .001) and OS (36.1 vs 12.4 months; HR, 0.46; 95% CI, 0.37-0.59; *P* < .001) compared with those with a low TMB (Figure 1), even after excluding *EGFR*-positive and *ALK-*positive NSCLCs, as well as never smokers (eFigure 14 in the Supplement). Baseline clinicopathological features of patients with low and high TMB in the combined cohort are summarized in Table 1. To further validate our findings, we performed meta-analyses of the combined cohorts that confirmed an association between high TMB and ORR (adjusted OR, 2.90; 95% CI, 1.78-4.70; *P* < .001), PFS (adjusted HR, 0.47; 95% CI, 0.36-0.61; *P* < .001), and OS (adjusted HR, 0.59; 95% CI, 0.44-0.79; *P* = .001) (eTable 7 in the Supplement).

**Table 1.** Characteristics of Patients With Non–Small Cell Lung Cancer Treated With PD-1/PD-L1 Inhibition in the Pooled Cohort of Patients From the Memorial Sloan Kettering Cancer Center, Dana-Farber Cancer Institute, and Stand Up To Cancer/Mark Foundation Data Sets, According to TMB Status

Clinical characteristic	Patients, No. (%)	
	Low TMB (n = 1391)	High TMB (n = 161)
Age, median (range), y	67 (22-92)	65 (44-83)
Sex		
Male	639 (45.9)	83 (51.6)
Female	752 (54.1)	78 (48.4)
Smoking status		
Current or former	1165 (83.8)	157 (97.5)
Never	226 (16.2)	4 (2.5)
Histologic profile		
Nonsquamous	1198 (86.1)	149 (92.5)
Squamous	193 (13.9)	12 (7.5)
Oncogenic driver mutation		
*KRAS*	482 (34.7)	38 (23.6)
*EGFR*	138 (9.9)	5 (3.1)
Other	120 (8.6)	15 (9.3)
None identified	651 (46.8)	103 (64.0)
Eastern Cooperative Oncology Group performance status		
0-1	1058 (85.5)	127 (89.4)
≥2	179 (14.5)	15 (10.6)
Not assessed	154 (NA)	19 (NA)
Line of therapy		
First	473 (34.0)	58 (36.0)
Second or higher	918 (66.0)	103 (64.0)
PD-L1 expression		
<1%	251 (27.4)	30 (28.3)
1%-49%	257 (28.1)	30 (28.3)
≥50%	407 (44.5)	46 (43.4)
Not assessed	476 (NA)	55 (NA)

Abbreviations: NA, not applicable; PD-1, programmed cell death–1; PD-L1, programmed death ligand 1; TMB, tumor mutation burden.

Previous studies^14,15^ have shown that gradually increasing TMB levels are associated with progressively improving clinical outcomes of ICI across different tumor types, suggesting a more continuous association of TMB with ICI efficacy. We also noted that ORR, PFS, and OS progressively improved along with increasing TMB percentile cutoffs (eFigures 15A, 15B, and 15C in the Supplement). As this gradual improvement in outcomes could be influenced by TMB outliers, we examined the response rate and the HRs for PFS and OS in each TMB decile independently, relative to the lowest decile as reference. Only patients with a TMB at the uppermost percentiles had improved ORR, PFS, and OS after receiving immunotherapy (eFigures 15A, 15D, and 15E in the Supplement), again suggesting that the benefit observed with increasing TMB cutoffs is associated primarily with NSCLCs with a very high TMB. To dissect characteristics of a very high TMB, we examined the impact of the same TMB threshold on a separate cohort of 1617 patients at DFCI and MSKCC with advanced NSCLC who never received ICI, and found that a high TMB (TMB *z* score >1.16, corresponding to a TMB >19.0 mutations per megabase in the MSKCC cohort and >19.3 mutations per megabase in the DFCI cohort) was not associated with the outcomes (eFigure 16 in the Supplement).

### TMB as a Biomarker of Response to PD-1/PD-L1 Blockade in the Context of Different PD-L1 Expression Levels

To investigate the differing associations of TMB and PD-L1 expression with clinical outcomes of ICI in the combined cohort, we examined the association of the high vs low TMB threshold with ORR, PFS, and OS to ICI across the 3 clinically relevant PD-L1 expression subgroups of less than 1%, 1% to 49%, and 50% or higher, as distinct PD-L1–based therapies have been approved on the basis of these PD-L1 categories.^1,2,3,4^ We identified that a high TMB (TMB *z* score >1.16 for each cohort) was associated with improved ORR and survival in each PD-L1 subset (eFigure 17 in the Supplement and Table 2), compared with a low TMB (TMB *z* score ≤1.16). Notably, patients with NSCLCs harboring both high TMB and PD-L1 expression 50% or higher experienced an ORR of 57% and also had the longest PFS (18.1 months) and OS (47.7 months) with ICI. In contrast, patients with low-TMB and PD-L1–negative NSCLC had the lowest ORR (8.7%) and the shortest PFS (2.1 months) and OS (10.4 months). These data indicate that TMB can further stratify outcomes of immunotherapy for patients within each clinically relevant PD-L1 expression group.

**Table 2.** Objective Response Rate, Progression-Free, and Overall Survival to PD-1/PD-L1 Blockade in High and Low TMB Non–Small Cell Lung Cancer According to PD-L1 Expression Subgroups

Outcome and PD-L1 tumor proportion score	Low TMB	High TMB	*P* value
Objective response rate, % (95% CI)			
<1%	8.7 (5.5-12.9)	46.7 (28.3-65.7)	<.001
1%-49%	18.7 (14.1-23.9)	50.0 (31.3-68.7)	<.001
≥50%	38.1 (33.3-43.0)	56.5 (41.1-71.1)	.02
Progression-free survival, median (95% CI), mo			
<1%	2.1 (2.0-2.4)	10.7 (8.2-24.4)	<.001
1%-49%	2.9 (2.5-3.6)	13.6 (8.6-NR)	<.001
≥50%	5.2 (4.6-6.2)	18.1 (8.6-NR)	<.001
Overall survival, median (95% CI), mo			
<1%	10.4 (7.9-13.6)	23.9 (16.7-NR)	.07
1%-49%	11.3 (9.6-14.7)	NR (21.2-NR)	<.001
≥50%	21.4 (17.5-25.9)	47.7 (35.4-NR)	.02

Abbreviations: NR, not reached; PD-1, programmed cell death–1; PD-L1, programmed death ligand–1; TMB, tumor mutation burden.

### Elevated TMB and Increased CD8-Positive PD1-Positive T Cells in NSCLC

**Figure 2.**
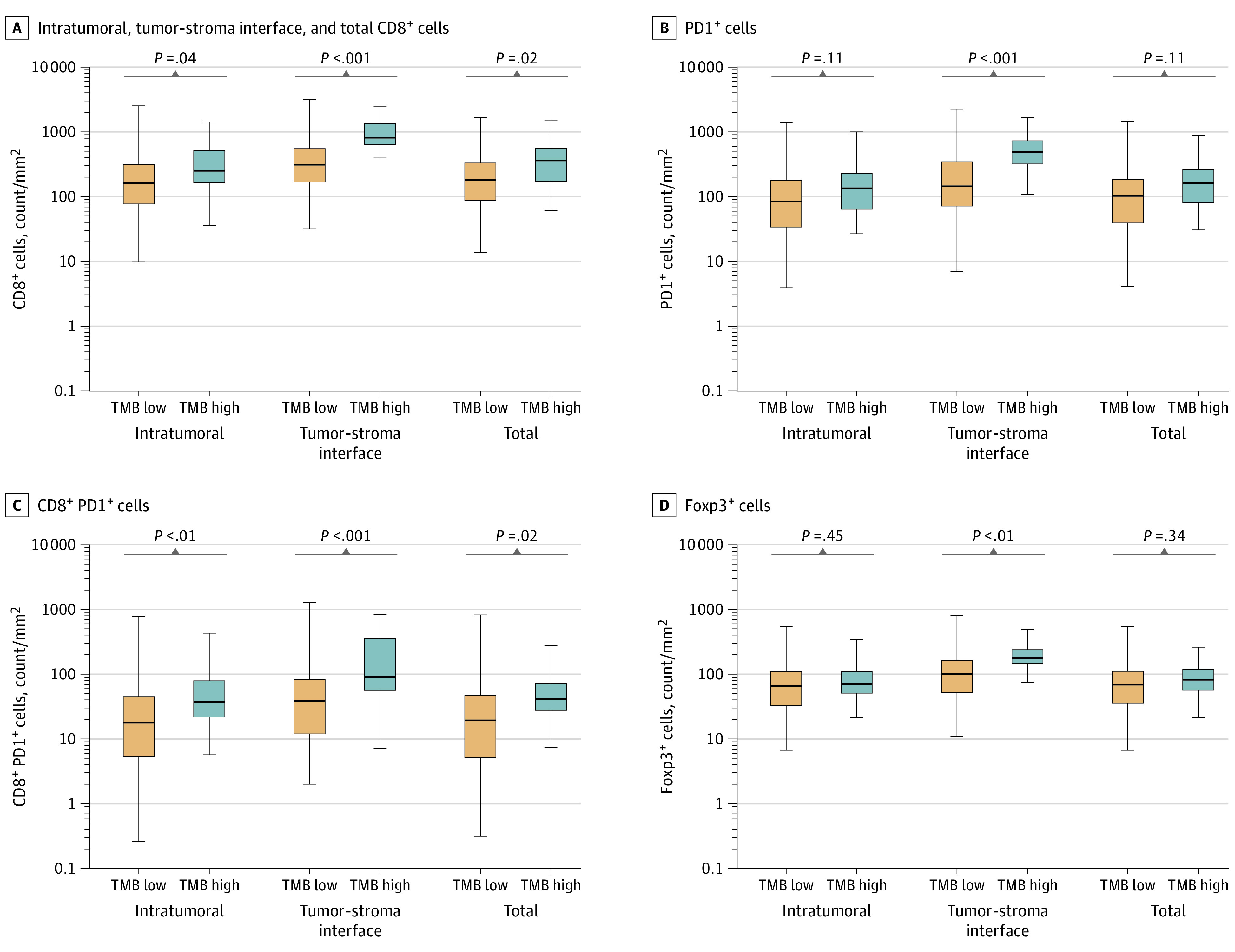
Multiplexed Immunofluorescence (ImmunoProfile) Showing Intratumoral, Tumor-Stroma Interface, and Total CD8^+^ Cells, PD1^+^ Cells, CD8^+^ PD1^+^ Cells, and Foxp3^+^ Cells in Patients With Non–Small Cell Lung Cancer Data are from patients in the Dana-Farber Cancer Institute cohort, including 384 patients with low tumor mutation burden (TMB) and 44 patients with high TMB.

To explore mechanisms by which NSCLCs with high TMB are more responsive to ICI, we next performed multiplexed immunofluorescence for CD8, Foxp3, PD-1, and PD-L1 on 428 NSCLC samples at DFCI. We found a significant association between higher TMB levels and increased CD8-positive T-cell counts intratumorally, at the tumor-stroma interface, and in total (Figure 2A); increased PD-1-positive cells at the tumor-stroma interface (Figure 2B); and increased CD8-positive, PD-1-positive T cells intratumorally, at the tumor-stroma interface, and in total (Figure 2C). No significant differences in intratumoral and total Foxp3-positive cells were identified in high-TMB vs low-TMB cancers (Figure 2D). Tumors with high TMB had also increased proportion of tumor cell, immune cell, and total PD-L1-positive cells (eFigure 18 in the Supplement). The linear association between TMB and CD8-positive, Foxp3-positive, PD-1-positive, and PD-L1-positive cells by multiplexed immunofluorescence is shown in eFigures 19 and 20 in the Supplement. Multiplexed immunofluorescence images from 3 representative high-TMB cases and 3 low-TMB low cases are shown in eFigure 21 in the Supplement.

To validate this finding in an independent external cohort, we deconvoluted RNaseq data from the The Cancer Genome Atlas NSCLC data set into tumor-associated cell populations using xCell software (2017 release; Institute for Computational Health Sciences, University of California, San Francisco)^21^ and identified that tumors with high TMB had a significantly higher proportion of CD8-positive T central memory cells, M1 macrophages, plasmacytoid dendritic cells, and Th1/Th2 T cells (eFigure 22 in the Supplement).

### TMB Levels and Distinct Mutational Patterns and Transcriptomic Profiles in NSCLC

Next, we examined whether these TMB subgroups had different mutational (DFCI) and transcriptomic (The Cancer Genome Atlas) profiles to identify additional factors that may affect tumor immunogenicity. Because nonsquamous and squamous NSCLCs have different genomic profiles and mutation patterns,^22,23^ we analyzed these tumors separately. The distribution of the most common mutations in each TMB group are shown in eFigure 23 in the Supplement (nonsquamous NSCLC) and eFigure 24 in the Supplement (squamous NSCLC). Compared with low-TMB nonsquamous NSCLCs, high-TMB tumors were significantly enriched for mutations in *TP53*, *KEAP1*, *BRAF*, and DNA damage repair genes (*ATM*, *BRCA1*, *BRCA2*, *ATR*, and *MSH2*), whereas *EGFR* mutation was enriched among low-TMB tumors (*Q* < 0.05) (eFigure 25A in the Supplement). *KRAS* and *STK11* had a similar prevalence in high vs low TMB nonsquamous NSCLCs. Among squamous cases, those with high TMB were also enriched for mutations in DNA damage repair genes, such as *ATM* and *BRCA1* (*Q* < 0.05) (eFigure 25B in the Supplement). Comutation analysis among high-TMB nonsquamous NSCLCs showed that *KRAS* and *KEAP1* mutations tended to be mutually exclusive, whereas there was no significant co-occurrence of *KRAS/STK11* mutations, in contrast to the low-TMB cohort, in which *KRAS* mutations significantly co-occurred with *STK11* and *KEAP1* (eFigure 26 in the Supplement). This observation is of interest given that concurrent *KRAS/STK11* and *KRAS/KEAP1* comutations have been shown to be associated with resistance to ICI in NSCLC.^24,25^ Comutation patterns in high and low TMB cases among squamous cancers are shown in eFigure 27 in the Supplement. These findings were also validated in an independent cohort of 915 nonsquamous NSCLCs sequenced by the MSK-IMPACT platform^18^ (eFigure 28 in the Supplement).

We next examined the relative contribution of transversion and transition mutations to the mutational load in each TMB grouping in the DFCI next-generation sequencing cohort, as this was previously shown to be associated with outcomes of ICI.^13^ The proportion of transversions was highest among tumors with high TMB (eFigure 29A in the Supplement). By contrast, tumors with low TMB had the highest proportion of transitions, likely reflecting differences in tobacco exposure (eFigure 29B in the Supplement). Within the group of high-TMB NSCLCs treated with immunotherapy at DFCI, a high transversion-transition ratio greater than or equal to 1 could further identify patients with improved PFS (HR, 0.41; 95% CI, 0.18-0.91; *P* = .03) and OS (HR, 0.44; 95% CI, 0.20-0.97; *P* = .04) after ICI, compared with a low transversion-transition ratio (eFigure 30A in the Supplement). Among tumors with low TMB, a high transversion-transition ratio was associated with improved PFS (HR, 0.81; 95% CI, 0.67-0.97; *P* = .02), but not with OS (HR, 0.85; 95% CI, 0.69-1.04; *P* = .12) (eFigure 30B in the Supplement).

Finally, we examined whether NSCLCs with different TMB levels demonstrated different transcriptomic profiles using the Cancer Genome Atlas lung adenocarcinoma and lung squamous cell carcinoma data sets. Compared with low-TMB cases, lung adenocarcinomas with high TMB showed marked upregulation of major histocompatibility complex class II antigen presentation and interleukin-7 signaling pathways, gene signatures of activated CD8-positive effector T cells and CD8a dendritic cells, and pathways involved in DNA repair (eg, DNA double-strand break response, base excision repair, and homologous recombination) (eFigure 31A in the Supplement). Similarly, lung squamous tumors with high TMB had a significant upregulation of pathways involved in antigen processing and presentation, chemokine signaling, and natural killer–cell mediated cytotoxicity, when compared with low-TMB cases (eFigure 31B in the Supplement). These findings indicate that in NSCLC, high levels of TMB are associated with increased immune cell infiltration and favorable transcriptomic profiles, which may enhance sensitivity to ICI.

## Discussion

Several studies^8,13,14,16^ have shown that a higher TMB generally is associated with clinical benefit from ICI. However, since there is variability among sequencing platforms as well as the cutoffs used to define what is considered to be a high TMB value, TMB alone is still not routinely used in NSCLC to make treatment decisions.^13,14,26^ In this cohort study, we found that patients with high TMB levels (approximately at the 90th percentile) derived the greatest improvement in terms of response to treatment and survival. Importantly, we extended this observation to PD-L1–negative and PD-L1–positive cases, indicating that TMB is a biomarker of the benefit from immunotherapies across all PD-L1 expression levels.

Several of our findings may explain the mechanistic association between a high TMB and improved clinical outcomes, including higher proportions of tumor-infiltrating, CD8-positive, PD1-positive, T cells, increased PD-L1 tumor expression, upregulation of pathways involved in innate and adaptative immune response, including major histocompatibility complex class II antigen presentation, and a distinct mutational landscape. Importantly, within the subset of patients with high TMB, those with a low transversion-transition ratio experienced significantly shorter survival compared with those with a high ratio, suggesting that the relative contribution of transversions and transitions may be used to identify patients with high TMB who may not respond to immunotherapy.

Consistent with our findings, a recent analysis^7^ of 1662 patients with advanced cancers treated with ICI showed that higher somatic TMB (highest 20% in each histologic grade) was associated with better OS. However, in that study, there was variation by cancer type in whether TMB levels were associated with improved survival after receipt of ICIs, highlighting the need determine how to optimally integrate TMB and other potential biomarkers of response to ICI within specific tumor types. In NSCLC, increasing PD-L1 expression levels on tumor cells generally are associated with response to immunotherapy.^2,5^ However, how to integrate TMB and PD-L1 expression to identify likely responders to ICI has been unclear. Previous studies^8,18^ exploring a cutoff of 10 mutations per megabase failed to show a significant impact on OS, and our results indicate that a higher TMB threshold closer to the 90th percentile may be necessary to clearly distinguish patients most likely to benefit from immunotherapy.

Here, using the power of a large cohort of immunotherapy-treated patients, which was possible only through a harmonized analysis across different sequencing platforms, we found that high TMB levels were associated with improved ICI efficacy across different PD-L1 expression subgroups, which has important implications. For patients with advanced NSCLC and a PD-L1 tumor proportion score of 1% or higher and 50% or higher, 2 therapeutic regimens are approved for use: ICI alone or in combination with chemotherapy.^1,2,4,27^ Because there are no prospective data comparing ICI alone with ICI plus chemotherapy, our results suggest that for patients with PD-L1 tumor proportion score of 1% to 49% or 50% or higher, and very high TMB, ICI may be a reasonable treatment option as monotherapy, sparing the potential toxicities of adding chemotherapy. As high TMB is a robust and independent biomarker of response to ICI, our data also suggest that TMB should routinely be introduced as a stratification factor for immunotherapy clinical trials, to ensure that outcomes are associated with treatment interventions, rather than imbalances in TMB distributions. Importantly, because genomic coverage can differ across sequencing platforms, these trials should use assays that provide at least 0.5 Mb, or optimally 0.8 Mb or more of coverage for sufficient and accurate TMB assessment.

### Limitations

A limitation of this study is its retrospective design. Another limitation is the lack of PD-L1 expression data for a fraction of patients.

## Conclusions

The findings of this cohort study suggest that TMB determination using next-generation sequencing may be a valuable biomarker for estimating immune checkpoint inhibitor efficacy. In addition, integration of TMB with PD-L1 expression may identify patients with the greatest likelihood of response to immunotherapy.

## Notes

Supplement. **eAppendix.** Supplemental Methods **eFigure 1. **Statistical Approach for the Determination and Validation of Tumor Mutational Burden Optimal Cut-Point in this Study **eFigure 2.** (A) Tumor Mutational Burden of 3591 NSCLCs Which Underwent Next-Generation Sequencing at the Dana-Farber Cancer Institute, Correlation Between TMB With (B) Tobacco History, (C) Number of Tobacco Pack-Years, (D) Tumor Histology, and (E) NSCLC Stage at the Time of Next-Generation Sequencing **eFigure 3.** (A) TMB Distributions According to NSCLC Genotype and (B) Q Values for Pairwise Comparisons of Tumor Genotype in Comparison to One Another (Benjamini-Hochberg Procedure) **eFigure 4.** Box Plot Showing the Distribution of TMB Among Patients With Non-Small Cell Lung Cancer Who Experienced a Complete/Partial Response, Stable Disease, and Progressive Disease as Best Response to PD-(L)1 Inhibition in the MSKCC, DFCI, and SU2C/Mark Foundation Cohorts **eFigure 5.** Normalization and Standardization of TMB Distributions Bring the Next-Generation Sequencing (MSK-IMPACT and DFCI OncoPanel) and WES Cohort (SU2C/Mark Foundation) Distributions Into Alignment **eFigure 6.** (A) Unbiased Regression Tree Modeling the Objective Response to PD-(L)1 Blockade as Function of Tumor Mutational Burden Identified an Optimal Threshold of 19.0 Mutations/Megabase That Discriminates Responders Versus Nonresponders in the MSKCC Discovery Cohort, (B) Objective Response Rate, (C) Progression-Free, and (D) Overall Survival to Immunotherapy in Patients With TMB-High (>19 mut/Mb) vs TMB-Low (≤19 mut/Mb) NSCLC in the MSKCC Discovery Cohort **eFigure 7.** (A) Objective Response Rate, (B) Progression-Free, and (C) Overall Survival to Immunotherapy in the DFCI Cohort According to TMB-High (>19.3 mut/Mb/TMB Z-Score >1.16) Versus TMB-Low TMB (≤19.3 mut/Mb/TMB Z-Score ≤1.16), (D) Objective Response Rate, (E) Progression-Free, and (F) Overall Survival to Immunotherapy According to TMB-High (>16.0 mut/Mb/TMB Z-Score >1.16) Versus TMB-Low TMB (≤16.0 mut/Mb/TMB Z-Score ≤1.16) Versus Low Harmonized TMB **eFigure 8.** Multivariable Analysis for Response, Progression-Free, and Overall Survival to PD-(L)1 Blockade in the Memorial Sloan Kettering Cancer Center Cohort **eFigure 9.** Multivariable Analysis for Response, Progression-Free, and Overall Survival to PD-(L)1 Blockade in the Dana-Farber Cancer Institute Cohort **eFigure 10.** Multivariable Analysis for Response, Progression-Free, and Overall Survival to PD-(L)1 Blockade in the Stand Up 2 Cancer/Mark Foundation Cohort **eFigure 11.** Multivariable Analysis With Inverse Probability Weighting for PD-L1 Expression for Response, Progression-Free, and Overall Survival to PD-(L)1 Blockade in the Memorial Sloan Kettering Cancer Center Cohort **eFigure 12.** Multivariable Analysis With Inverse Probability Weighting for PD-L1 Expression for Response, Progression-Free, and Overall Survival to PD-(L)1 Blockade in the Dana-Farber Cancer Institute Cohort **eFigure 13.** Multivariable Analysis With Inverse Probability Weighting for PD-L1 Expression for Response, Progression-Free, and Overall Survival to PD-(L)1 Blockade in the Stand Up 2 Cancer/Mark Foundation Cohort **eFigure 14.** (A) Objective Response Rate, (B) Progression-Free Survival, and (C) Overall Survival in Patients With High Versus Low Harmonized TMB in the Pooled Cohort of NSCLCs Treated With PD-(L)1 Blockade From DFCI, MSKCC, and the SU2C/Mark Foundation Dataset, After Excluding EGFR and ALK Positive Cases **eFigure 15.** Objective Response Rate by Increasing TMB Percentiles Thresholds (Upper Panel), and in Each TMB Decile (Lower Panel) in the Combined Cohort **eFigure 16.** Overall Survival in Patients at DFCI and MSKCC With Advanced Non-Small Cell Lung Cancer Who Never Received Immunotherapy According to TMB Levels **eFigure 17.** (A) Response Rate, (B) Progression-Free, and (C) Overall Survival to PD-(L)1 Inhibition According to TMB Levels Among Non-Small Cell Lung Cancers With a PD-L1 TPS <1% **eFigure 18.** Percentage of Tumor, Immune, and Total Cells With PD-L1 Expression Among NSCLC Samples With Low (N = 384) and High (N = 44) TMB Which Also Underwent Multiplexed Immunofluorescence at the DFCI **eFigure 19.** Linear Correlation Between TMB and CD8+, PD-1+, CD8+ PD-1+, and Foxp3+Cells Intratumorally (A) and at the Tumor-Stroma Interface (B) Among 428 NSCLCs at DFCI Which Underwent Multiplexed Immunofluorescence **eFigure 20.** Linear Correlation Between TMB and (A) Total CD8+, PD-1+, CD8+ PD-1+, and Foxp3+ Cells, and (B) Linear Correlation Between Tumoral, Immune, and total PD-L1+ Cells Among 428 NSCLCs at DFCI Which Underwent Multiplexed Immunofluorescence **eFigure 21.** Multiplexed Immunofluorescence for CD8, PD-1, Foxp3, PD-L1, in Three Index Cases With High TMB (A) and Three Index Cases With Low TMB (B) **eFigure 22.** Deconvolution of RNAseq Data From the NSCLC TCGA Dataset (N=998) Into Tumor-Associated Immune Cells, Showing Cell Types That Are Significantly Enriched in NSCLCs With High vs Low TMB **eFigure 23.** OncoPrint Plot Showing the Top 20 Mutated Genes in 3168 Nonsquamous NSCLCs With High and Low TMB in the DFCI Genomic Cohort **eFigure 24.** OncoPrint Plot Showing the Top 20 Mutated Genes in 409 Squamous NSCLCs With High and Low TMB in the DFCI Genomic Cohort **eFigure 25.** Volcano Plot Showing Gene Mutations Enriched in TMB High Versus TMB Low (A) Nonsquamous (N=3168) and (B) Squamous (N=409) Non-Small Cell Lung Cancers in the DFCI Genomic Cohort **eFigure 26.** Comutation Patterns Among Lung Non-Squamous Carcinomas With (A) High TMB (N=365) and (B) Low TMB (N=2803) in the DFCI Genomic Cohort **eFigure 27.** Comutation Patterns Among Lung Squamous Carcinomas With (A) High TMB (N=39) and (B) Low TMB (N=370) in the DFCI Genomic Cohort **eFigure 28.** Volcano Plot Showing Gene Mutations Enriched in TMB High Versus TMB Low Nonsquamous NSCLC Among 915 Samples Which Underwent NGS at MSKCC **eFigure 29.** (A) Boxplot Showing Overall Distribution of Nucleotide Conversions and Stacked Barplot Showing Fraction of Conversions in Each NSCLC Sample With TMB High in the DFCI Genomic Cohort (N=404) and (B) Boxplot Showing Overall Distribution of Nucleotide Conversions and Stacked Barplot Showing Fraction of Conversions in Each NSCLC Sample With TMB Low in the DFCI Genomic Cohort (N=3173) **eFigure 30.** (A) Progression-Free and Overall Survival to PD-(L)1 Blockade Among Patients With High (≥1) Versus Low (<1) Transversion/Transition Ratio Among Patients With TMB High and (B) Progression-Free and Overall Survival to PD-(L)1 Blockade Among Patients With High (≥1) Versus Low (<1) Transversion/Transition Ratio Among Patients With TMB Low **eFigure 31.** Gene Set Enrichment Analysis Showing Prioritized Pathways Upregulated in TMB High Versus TMB Low NSCLC in (A) Lung Adenocarcinoma, and (B) Lung Squamous Carcinoma in the TCGA Cohort **eTable 1.** Clinicopathologic and Genomic Characteristics of the 3591 NSCLCs Which Underwent Next-Generation Sequencing at the Dana-Farber Cancer Institute **eTable 2.** Characteristics of Patients With NSCLC Treated With Immune Checkpoint Inhibitors at Memorial Sloan Kettering Cancer Center (MSKCC), Dana-Farber Cancer Institute (DFCI), and Stand Up To Cancer Foundation (SU2C)/Mark Foundation Dataset **eTable 3.** Tumor Mutational Burden (TMB) Values (in Mutations per Megabase, mut/Mb) at the Memorial Sloan Kettering Cancer Center (MSKCC), Dana-Farber Cancer Institute (DFCI), and Stand Up To Cancer/Mark Foundation (SU2C) Cohorts Which Correspond With the Harmonized TMB Z-Score of 1.16 **eTable 4.** Adjusted Odds Ratio for Response and Adjusted Hazard Ratio for Progression-Free and Overall Survival to PD-(L)1 Inhibition in the MSKCC Cohort After Multiple Imputation to Account for PD-L1 Missingness **eTable 5.** Adjusted Odds Ratio for Response and Adjusted Hazard Ratio for Progression-Free and Overall Survival to PD-(L)1 Inhibition in the DFCI Cohort After Multiple Imputation to Account for PD-L1 Missingness **eTable 6.** Adjusted Odds Ratios for Response and Adjusted Hazard Ratio for Progression-Free and Overall Survival to PD-(L)1 Inhibition in the SU2C/Mark Foundation Cohort After Multiple Imputation to Account for PD-L1 Missingness **eTable 7.** Impact of TMB High Versus Low on Objective Response, Progression-Free, and Overall Survival in a Meta-analysis of the MSKCC and DFCI Cohorts
eReferences
