# Supplementary material for: Association of High Tumor Mutation Burden in Non–Small Cell Lung Cancers With Increased Immune Infiltration and Improved Clinical Outcomes of PD-L1 Blockade Across PD-L1 Expression Levels
Source: JAMA Oncol. 2022 Jun 16;8(8):1160–8. doi: 10.1001/jamaoncol.2022.1981 (PMC9204620; doi:10.1001/jamaoncol.2022.1981)
Supplement: Supplement. — eAppendix. Supplemental Methods eFigure 1. Statistical Approach for the Determination and Validation of Tumor Mutational Burden Optimal Cut-Point in this Study eFigure 2. (A) Tumor Mutational Burden of 3591 NSCLCs Which Underwent Next-Generation Sequencing at the Dana-Farber Cancer Institute, Correlation Between TMB With (B) Tobacco History, (C) Number of Tobacco Pack-Years, (D) Tumor Histology, and (E) NSCLC Stage at the Time of Next-Generation Sequencing eFigure 3. (A) TMB Distributions According to NSCLC Genotype and (B) Q Values for Pairwise Comparisons of Tumor Genotype in Comparison to One Another (Benjamini-Hochberg Procedure) eFigure 4. Box Plot Showing the Distribution of TMB Among Patients With Non-Small Cell Lung Cancer Who Experienced a Complete/Partial Response, Stable Disease, and Progressive Disease as Best Response to PD-(L)1 Inhibition in the MSKCC, DFCI, and SU2C/Mark Foundation Cohorts eFigure 5. Normalization and Standardization of TMB Distributions Bring the Next-Generation Sequencing (MSK-IMPACT and DFCI OncoPanel) and WES Cohort (SU2C/Mark Foundation) Distributions Into Alignment eFigure 6. (A) Unbiased Regression Tree Modeling the Objective Response to PD-(L)1 Blockade as Function of Tumor Mutational Burden Identified an Optimal Threshold of 19.0 Mutations/Megabase That Discriminates Responders Versus Nonresponders in the MSKCC Discovery Cohort, (B) Objective Response Rate, (C) Progression-Free, and (D) Overall Survival to Immunotherapy in Patients With TMB-High (>19 mut/Mb) vs TMB-Low (≤19 mut/Mb) NSCLC in the MSKCC Discovery Cohort eFigure 7. (A) Objective Response Rate, (B) Progression-Free, and (C) Overall Survival to Immunotherapy in the DFCI Cohort According to TMB-High (>19.3 mut/Mb/TMB Z-Score >1.16) Versus TMB-Low TMB (≤19.3 mut/Mb/TMB Z-Score ≤1.16), (D) Objective Response Rate, (E) Progression-Free, and (F) Overall Survival to Immunotherapy According to TMB-High (>16.0 mut/Mb/TMB Z-Score >1.16) Versus TMB-Low TMB (≤16.0 mut/Mb/TMB [file jamaoncol-e221981-s001.pdf]

Supplemental Online Content

Ricciuti B, Wang X, Alessi JV, et al. Association of high tumor mutation burden in non–small cell lung cancers with increased immune infiltration and improved clinical outcomes of PD-L1 blockade across PD-L1 expression levels. *JAMA Oncology*. Published online June 16, 2022. doi:10.1001/jamaoncol.2022.1981

eAppendix. Supplemental Methods

eFigure 1. Statistical Approach for the Determination and Validation of Tumor Mutational Burden Optimal Cut-Point in this Study

eFigure 2. (A) Tumor Mutational Burden of 3591 NSCLCs Which Underwent Next-Generation Sequencing at the Dana-Farber Cancer Institute, Correlation Between TMB With (B) Tobacco History, (C) Number of Tobacco Pack-Years, (D) Tumor Histology, and (**E**) NSCLC Stage at the Time of Next-Generation Sequencing

eFigure 3. (A) TMB Distributions According to NSCLC Genotype and (B) Q Values for Pairwise Comparisons of Tumor Genotype in Comparison to One Another (Benjamini-Hochberg Procedure)

eFigure 4. Box Plot Showing the Distribution of TMB Among Patients With Non-Small Cell Lung Cancer Who Experienced a Complete/Partial Response, Stable Disease, and Progressive Disease as Best Response to PD-(L)1 Inhibition in the MSKCC, DFCI, and SU2C/Mark Foundation Cohorts

eFigure 5. Normalization and Standardization of TMB Distributions Bring the Next-Generation Sequencing (MSK-IMPACT and DFCI OncoPanel) and WES Cohort (SU2C/Mark Foundation) Distributions Into Alignment

eFigure 6. (A) Unbiased Regression Tree Modeling the Objective Response to PD-(L)1 Blockade as Function of Tumor Mutational Burden Identified an Optimal Threshold of 19.0 Mutations/Megabase That Discriminates Responders Versus Nonresponders in the MSKCC Discovery Cohort, (B) Objective Response Rate, (C) Progression-Free, and (D) Overall Survival to Immunotherapy in Patients With TMB-High (>19 mut/Mb) vs TMB-Low (≤19 mut/Mb) NSCLC in the MSKCC Discovery Cohort

eFigure 7. (A) Objective Response Rate, (B) Progression-Free, and (C) Overall Survival to Immunotherapy in the DFCI Cohort According to TMB-High (>19.3 mut/Mb/TMB Z-Score >1.16) Versus TMB-Low TMB (≤19.3 mut/Mb/TMB Z-Score ≤1.16), (D) Objective Response Rate, (E) Progression-Free, and (F) Overall Survival to Immunotherapy According to TMB-High (>16.0 mut/Mb/TMB Z-Score >1.16) Versus TMB-Low TMB (≤16.0 mut/Mb/TMB Z-Score ≤1.16) Versus Low Harmonized TMB

eFigure 8. Multivariable Analysis for Response, Progression-Free, and Overall Survival to PD-(L)1 Blockade in the Memorial Sloan Kettering Cancer Center Cohort

eFigure 9. Multivariable Analysis for Response, Progression-Free, and Overall Survival to PD-(L)1 Blockade in the Dana-Farber Cancer Institute Cohort

eFigure 10. Multivariable Analysis for Response, Progression-Free, and Overall Survival to PD-(L)1 Blockade in the Stand Up 2 Cancer/Mark Foundation Cohort

eFigure 11. Multivariable Analysis With Inverse Probability Weighting for PD-L1 Expression for Response, Progression-Free, and Overall Survival to PD-(L)1 Blockade in the Memorial Sloan Kettering Cancer Center Cohort

eFigure 12. Multivariable Analysis With Inverse Probability Weighting for PD-L1 Expression for Response, Progression-Free, and Overall Survival to PD-(L)1 Blockade in the Dana-Farber Cancer Institute Cohort

© 2022 Ricciuti B et al. *JAMA Oncology.*

eFigure 13. Multivariable Analysis With Inverse Probability Weighting for PD-L1 Expression for Response, Progression-Free, and Overall Survival to PD-(L)1 Blockade in the Stand Up 2 Cancer/Mark Foundation Cohort

eFigure 14. (A) Objective Response Rate, (B) Progression-Free Survival, and (C) Overall Survival in Patients With High Versus Low Harmonized TMB in the Pooled Cohort of NSCLCs Treated With PD-(L)1 Blockade From DFCI, MSKCC, and the SU2C/Mark Foundation Dataset, After Excluding *EGFR* and *ALK* Positive Cases

eFigure 15. Objective Response Rate by Increasing TMB Percentiles Thresholds (Upper Panel), and in Each TMB Decile (Lower Panel) in the Combined Cohort

eFigure 16. Overall Survival in Patients at DFCI and MSKCC With Advanced Non-Small Cell Lung Cancer Who Never Received Immunotherapy According to TMB Levels

eFigure 17. (A) Response Rate, (B) Progression-Free, and (C) Overall Survival to PD-(L)1 Inhibition According to TMB Levels Among Non-Small Cell Lung Cancers With a PD-L1 TPS <1%

eFigure 18. Percentage of Tumor, Immune, and Total Cells With PD-L1 Expression Among NSCLC Samples With Low (N = 384) and High (N = 44) TMB Which Also Underwent Multiplexed Immunofluorescence at the DFCI

eFigure 19. Linear Correlation Between TMB and CD8$^+$, PD-1$^+$, CD8$^+$ PD-1$^+$, and Foxp3$^+$Cells Intratumorally (A) and at the Tumor-Stroma Interface (B) Among 428 NSCLCs at DFCI Which Underwent Multiplexed Immunofluorescence

eFigure 20. Linear Correlation Between TMB and (A) Total CD8$^+$, PD-1$^+$, CD8$^+$ PD-1$^+$, and Foxp3$^+$ Cells, and (B) Linear Correlation Between Tumoral, Immune, and total PD-L1$^+$ Cells Among 428 NSCLCs at DFCI Which Underwent Multiplexed Immunofluorescence

eFigure 21. Multiplexed Immunofluorescence for CD8, PD-1, Foxp3, PD-L1, in Three Index Cases With High TMB (A) and Three Index Cases With Low TMB (B)

eFigure 22. Deconvolution of RNAseq Data From the NSCLC TCGA Dataset (N=998) Into Tumor-Associated Immune Cells, Showing Cell Types That Are Significantly Enriched in NSCLCs With High vs Low TMB

eFigure 23. OncoPrint Plot Showing the Top 20 Mutated Genes in 3168 Nonsquamous NSCLCs With High and Low TMB in the DFCI Genomic Cohort

eFigure 24. OncoPrint Plot Showing the Top 20 Mutated Genes in 409 Squamous NSCLCs With High and Low TMB in the DFCI Genomic Cohort

eFigure 25. Volcano Plot Showing Gene Mutations Enriched in TMB High Versus TMB Low (A) Nonsquamous (N=3168) and (B) Squamous (N=409) Non-Small Cell Lung Cancers in the DFCI Genomic Cohort

eFigure 26. Comutation Patterns Among Lung Non-Squamous Carcinomas With (A) High TMB (N=365) and (B) Low TMB (N=2803) in the DFCI Genomic Cohort

eFigure 27. Comutation Patterns Among Lung Squamous Carcinomas With (A) High TMB (N=39) and (B) Low TMB (N=370) in the DFCI Genomic Cohort

eFigure 28. Volcano Plot Showing Gene Mutations Enriched in TMB High Versus TMB Low Nonsquamous NSCLC Among 915 Samples Which Underwent NGS at MSKCC

eFigure 29. (A) Boxplot Showing Overall Distribution of Nucleotide Conversions and Stacked Barplot Showing Fraction of Conversions in Each NSCLC Sample With TMB High in the DFCI Genomic Cohort (N=404) and (B) Boxplot Showing Overall Distribution of Nucleotide Conversions and Stacked Barplot Showing Fraction of Conversions in Each NSCLC Sample With TMB Low in the DFCI Genomic Cohort (N=3173)

eFigure 30. (A) Progression-Free and Overall Survival to PD-(L)1 Blockade Among Patients With High (≥1) Versus Low (<1) Transversion/Transition Ratio Among Patients With TMB High and (B) Progression-Free and Overall Survival to PD-(L)1 Blockade Among Patients With High (≥1) Versus Low (<1) Transversion/Transition Ratio Among Patients With TMB Low

© 2022 Ricciuti B et al. *JAMA Oncology.*

eFigure 31. Gene Set Enrichment Analysis Showing Prioritized Pathways Upregulated in TMB High Versus TMB Low NSCLC in (A) Lung Adenocarcinoma, and (B) Lung Squamous Carcinoma in the TCGA Cohort

eTable 1. Clinicopathologic and Genomic Characteristics of the 3591 NSCLCs Which Underwent Next-Generation Sequencing at the Dana-Farber Cancer Institute

eTable 2. Characteristics of Patients With NSCLC Treated With Immune Checkpoint Inhibitors at Memorial Sloan Kettering Cancer Center (MSKCC), Dana-Farber Cancer Institute (DFCI), and Stand Up To Cancer Foundation (SU2C)/Mark Foundation Dataset

eTable 3. Tumor Mutational Burden (TMB) Values (in Mutations per Megabase, mut/Mb) at the Memorial Sloan Kettering Cancer Center (MSKCC), Dana-Farber Cancer Institute (DFCI), and Stand up To Cancer/Mark Foundation (SU2C) Cohorts Which Correspond With the Harmonized TMB Z-Score of 1.16

eTable 4. Adjusted Odds Ratio for Response and Adjusted Hazard Ratio for Progression-Free and Overall Survival to PD-(L)1 Inhibition in the MSKCC Cohort After Multiple Imputation to Account for PD-L1 Missingness

eTable 5. Adjusted Odds Ratio for Response and Adjusted Hazard Ratio for Progression-Free and Overall Survival to PD-(L)1 Inhibition in the DFCI Cohort After Multiple Imputation to Account for PD-L1 Missingness

eTable 6. Adjusted Odds Ratios for Response and Adjusted Hazard Ratio for Progression-Free and Overall Survival to PD-(L)1 Inhibition in the SU2C/Mark Foundation Cohort After Multiple Imputation to Account for PD-L1 Missingness

eTable 7. Impact of TMB High Versus Low on Objective Response, Progression-Free, and Overall Survival in a Meta-analysis of the MSKCC and DFCI Cohorts

eReferences

This supplemental material has been provided by the authors to give readers additional information about their work.

© 2022 Ricciuti B et al. *JAMA Oncology.*

eAppendix. Supplemental Methods

Patient population

Dana-Farber Cancer Institute cohort

Patients at the Dana-Farber Cancer Institute who consented to institutional review board-approved protocols DF/HCC 02-180, 11-104, 13-364, and/or 17-000 which allowed for conducting translational research and tumor next-generation sequencing, respectively, were included.

Memorial Sloan Kettering Cancer Center cohort

Patients at the Memorial Sloan Kettering Cancer Center were included if they had advanced NSCLC which underwent tumor next generation sequencing and if they had also consented to institutional review board-approved protocols.

Stand Up to Cancer/Mark Foundation

Patients included in the Stand Up to Cancer/Mark Foundation cohort were enrolled if they had advanced NSCLC which was treated with PD-1/PD-L1 inhibitors.

Programmed death ligand 1 immunohistochemistry

The PD-L1 tumor proportion score (TPS) was determined by immunohistochemistry using validated anti-PD-L1 antibodies: E1L3N (Cell Signaling Technology, Danvers, MA), 22C3 (Dako North America Inc, Carpinteria, CA), 28-8 (Epitomics Inc, Burlingame, CA), according to local institutional practice.

Tumor genomic profiling and somatic variant calling in the DFCI and MSKCC cohorts

Tumor genomic profiling and somatic variants were performed using clinically validated bioinformatics pipelines[1,2]. Sequence reads were aligned to reference sequence b37 edition from the Human Genome Reference Consortium using bwa (http://bio-bwa.sourceforge.net/bwa.shtml), and further processed using Picard (version 1.90, http://broadinstitute.github.io/picard/) to remove duplicates and Genome Analysis Toolkit (GATK) to perform localized realignment around indel sites. Single nucleotide variants were called using MuTect v1.1.4, insertions and deletions were called using GATK Indelocator, and variants were annotated using Oncotator. In the DFCI cohort, to filter out potential germline variants, the standard pipeline removed SNPs present at >0.1% in Exome Variant Server, NHLBI GO Exome Sequencing Project (ESP) (URL: http://evs.gs.washington.edu/EVS/), present in dbSNP, or present in an in-house panel of normals, but rescues those also present in the COSMIC database. For this study, variants were further filtered by removing variants present at >0.1% in the gnomAD v.2.1.1 database or were annotated as Benign or Likely Benign in the ClinVar database (PMID: 32461654, 29165669). In the MSKCC cohort, patient-matched normal DNA was used to filter out germline variants, as previously described.

Tumor mutational burden assessment

DFCI OncoPanel and MSK-IMPACT

Tumor mutational burden (TMB), defined as the number of somatic, coding, base substitution, and indel mutations per megabase (Mb) of genome examined, was determined using the OncoPanel (Dana-Faber) and MSK-IMPACT (MSKCC) NGS platforms, as previously described[1,2]. DFCI mutation counts were divided by the number of bases covered in each OncoPanel version: v1, 0.753334 Mb; v2, 0.826167 Mb; and v3, 1.315078 Mb. For MSKCC samples, the mutation count was divided by 0.896665, 1.016478, and 1.139322 Mb for the 341-, 410-, and 468-gene panels, respectively.

Stand Up to Cancer cohort whole exome sequencing

DNA was extracted from FFPE tumor specimens and either matched normal whole blood or in cases where this was unavailable, adjacent normal FFPE specimens. Extraction was performed using the Qiagen AllPrep DNA/RNA Mini Kit (cat# 80204). A single aliquot of 150-500 ng input DNA in 100 µl TE buffer was used for library generation. Library preparation was performed using the Kapa HyperPrep kit, and quantification was performed using PicoGreen. Adapter ligation was performed

© 2022 Ricciuti B et al. *JAMA Oncology.*

using the TruSeq DNA exome kit from Illumina per manufacturer's instructions. Sequencing of pooled libraries was performed using a HiSeq2500 with 76 bp paired end reads. Mean target coverage for tumor and normal samples were 150X and 80X, respectively. Tumor mutational burden was defined as the number of non-synonymous base substitutions, indel mutations per megabase of genome examined, using an exome size of 35.8 Mb.

Tumor mutational burden normalization across different platforms

TMB distributions were harmonized between the two platforms by applying a normal transformation followed by standardization to Z-scores, as previously described[3]. Briefly, power transformations were first used to normalize cohort-specific TMB distributions; second, Tukey's ladder of powers in the rcompanion package was used to identify the optimal transformation coefficient. Third, the normalized distributions were then standardized into z scores by subtracting the transformed distribution mean and dividing by the standard deviation.

Cell subset analysis from the TCGA dataset

To perform cell type enrichment analyses, RNA sequencing data from the LUAD and LUSC TCGA cohort were deconvoluted to estimate cell subsets using the xCell package. xCell estimates the abundance scores of 64 cell types, including adaptive and innate immune cells, hematopoietic progenitors, epithelial cells, and extracellular matrix cells, based on single sample gene set enrichment analysis (ssGSEA) data[4]. Gene expression values (RSEM V2) were converted into Z-scores and used to compute cell type enrichment scores with the xCellAnalysis function. Statistical significance of differential cell type enrichment between cohorts was estimated with Wilcox Rank Sum test. Cell subtypes examined included: aDC, Adipocytes, Astrocytes, B-cells, Basophils, CD4+ memory T-cells, CD4+ naive T-cells, CD4+ T-cells, CD4+ Tcm, CD4+ Tem, CD8+ naive T-cells, CD8+ T-cells, CD8+ Tcm, CD8+ Tem, cDC, Chondrocytes, Class-switched memory B-cells, CLP, CMP, DC, Endothelial cells, Eosinophils, Epithelial cells, Erythrocytes, Fibroblasts, GMP, Hepatocytes, HSC, iDC, Keratinocytes, Endothelial cells, Macrophages, Macrophages M1, Macrophages M2, Mast cells, Megakaryocytes, Melanocytes, Memory B-cells, MEP, Mesangial cells, Monocytes, MPP, MSC, Endothelial cells, Myocytes, naive B-cells, Neurons, Neutrophils, NK cells, NKT, Osteoblast, pDC, Pericytes, Plasma cells, Platelets, Preadipocytes, pro B-cells, Sebocytes, Skeletal muscle, Smooth muscle, Tgd cells, Th1 cells, Th2 cells, Tregs.

Multiplexed immunofluorescence (ImmunoProfile)

Multiplexed immunofluorescence (mIF) was performed on samples from the Dana-Farber Cancer Institute by staining 5-micron formalin-fixed, paraffin-embedded whole tissue sections with standard, primary antibodies sequentially and paired with a unique fluorochrome followed by staining with nuclear counterstain/4′,6-diamidino-2-phenylindole (DAPI), as previously described[5]. All samples were stained for PD-L1 (clone E1L3N), PD-1 [clone EPR4877(2)], CD8 (clone 4B11), FOXP3 (clone D608R), Cytokeratin (clone AE1/AE3), and DAPI (nuclear counterstain). Each sample had a single slide stained and scanned at 20x resolution by a Vectra Polaris imaging platform. Regions of Interest (ROIs) were defined for each image, and only these regions were used for quantitative image analysis currently. Within each ROI, InForm Image Analysis software (PerkinElmer/Akoya) was run to phenotype and score cells based on biomarker expression. A custom script quantified the number/percentage of cells which are positive for relevant biomarkers in specific tissue regions. Each ROI was divided into one or more of these defined regions: intra-tumoral (IT), which was defined as the region of the slide consisting of tumor beyond the tumor-stroma interface; tumor-stroma interface (TSI), which was defined as the region within 40 microns to either side of the defined border between tumor and stroma; and total (IT + TSI). Cell count was calculated per ROI and averaged (unweighted) across ROIs, reported as count per millimeter squared +/- standard error. Statistical significance of differential cell type enrichment between groups was estimated with Wilcox Rank Sum test.

Gene expression analysis

Gene expression data were downloaded from the Firehose website (TCGA Firehose Legacy version) while somatic mutation data were downloaded from cBioPortal website (cbioportal.org). The RSEM V2 values were used to represent gene expression and genes with counts less than 10 were filtered out. Gene expression profiles were analyzed according to TMB categories. Median expression within each group was used to estimate expression fold-change (FC) to minimize the possible impact of outlier samples. Gene differential expression analyses across TMB subgroups were conducted using R package DESeq2. P-values were corrected for multiple hypothesis testing via false discovery rate (FDR) adjustment. Fold-change threshold of an

© 2022 Ricciuti B et al. *JAMA Oncology.*

absolute value greater than 1.5 and FDR adjusted P-value threshold less than 0.1 were utilized to identify differentially expressed genes. Pathway enrichment analyses were conducted separately for up- and down-regulated genes using Molecular Signatures Database (MSigDB) collections.

Statistical analysis

Clinicopathologic data and immunotherapy response data were abstracted from the electronic medical record. Overall response rate was determined by a blinded radiologist using Response Evaluation Criteria In Solid Tumors, version (RECIST) 1.1. Progression-free survival was determined from the start date of immunotherapy until the date of disease progression or death, and overall survival was calculated from the date of diagnosis of advanced NSCLC until the date of death. All p-values are two-sided and confidence intervals are at the 95% level. Overall survival among patients who never received PD-(L)1 inhibition was calculated from the date of the start of systemic therapy for advanced disease, other than immunotherapy. TMB comparisons were computed using the Mann-Whitney U test or the Kruskal-Wallis test, when appropriate. Linear correlations were evaluated using Spearman's test, and categorical variables were evaluated using Fisher's exact test. Event-time distributions were estimated using Kaplan-Meier methodology. Log-rank tests were used to test for differences in event-time distributions, and Cox proportional hazards models were fitted to obtain estimates of hazard ratios in univariate and multivariate models. The proportional hazards assumption was assessed with Schoenfeld residuals. All P-values are 2-sided and confidence intervals are at the 95% level, with significance pre-defined to be at <0.05. Multiple comparison correction was performed using the Benjamini-Hochberg procedure. Missing values were handled using inverse probability weighting (IPW) and multiple imputation approaches using R package MICE, as previously described. All statistical analyses were performed using R version 3.6.3.

TMB cut-off identification and validation

To identify and validate TMB thresholds associated with immunotherapy efficacy, an unbiased recursive partitioning algorithm was used to investigate an optimal grouping of TMB with respect to the objective response rate to immune checkpoint inhibition in a discovery cohort comprised of patients from the MSKCC cohort, using the partykit function in R, as previously described[6]. A 10-fold cross-validation method was used to train and measure the performance of the model using the caret function in R, as previously described[7]. The threshold identified was validated in two independent cohorts of patients treated with PD-(L)1 blockade in the DFCI and SU2C/Mark Foundation cohorts, following TMB harmonization across platforms, as described above and as previously described[3]. As PD-L1 tumor proportion score (TPS) is an important predictor for ICI efficacy, we applied both Inverse probability weighting (IPW) and multiple imputation approaches using R package MICE to address the potential selection bias arising from the PD-L1 TPS missingness. Variables used for multiple imputation and to calculate the weights for PD-L1 TPS missingness included sex, age, ECOG performance status, histology, smoking status, and line of therapy for ICI. IPW and multiple imputation were conducted separately in each cohort, and the multivariable analyses results were pooled based on 5 repeated complete imputed datasets.

© 2022 Ricciuti B et al. *JAMA Oncology.*

eFigure 1

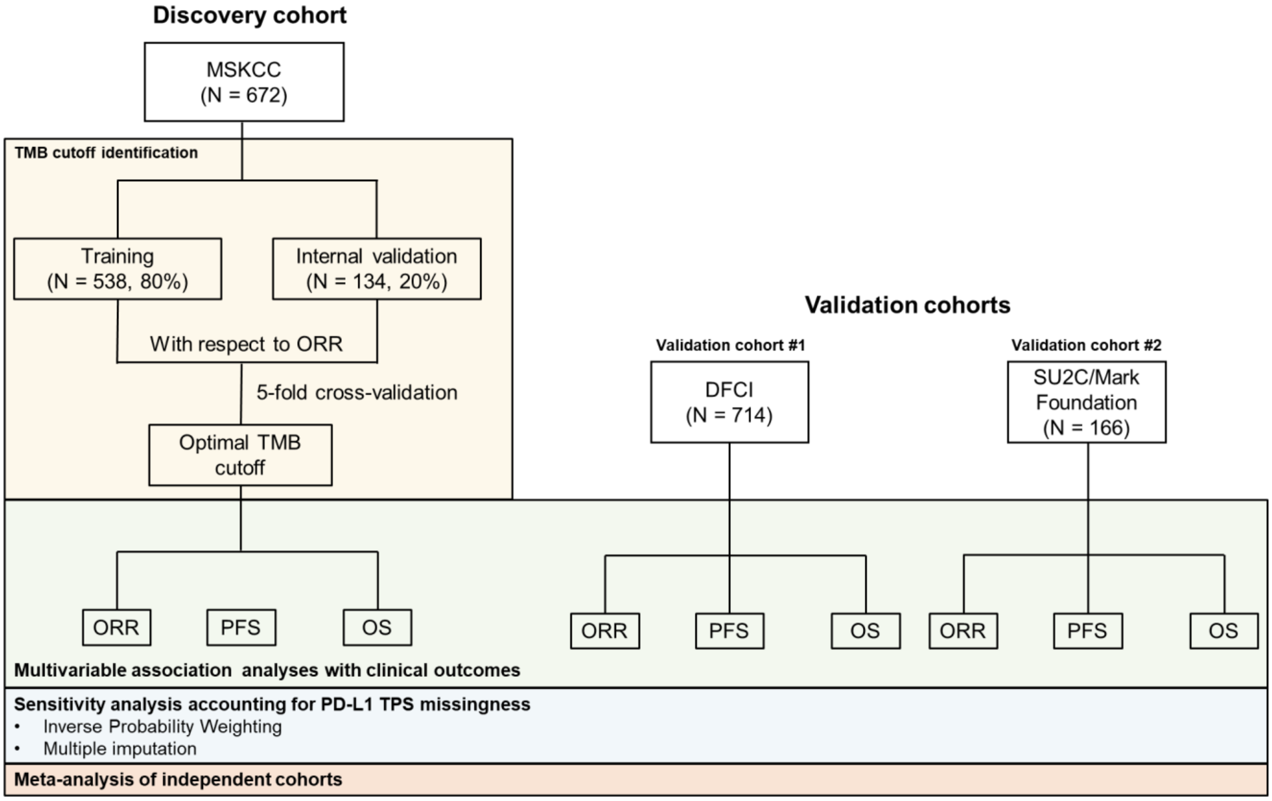

eFigure 1. Statistical Approach for the Determination and Validation of Tumor Mutational Burden Optimal Cut-Point in this Study. MSKCC, Memorial Sloan Kettering Cancer Center; DFCI, Dana-Farber Cancer Institute; SU2C, Stand Up To Cancer/Mark Foundation; ORR, objective response rate; PFS, progression-free survival; OS, overall survival.

© 2022 Ricciuti B et al. *JAMA Oncology.*

eFigure 2

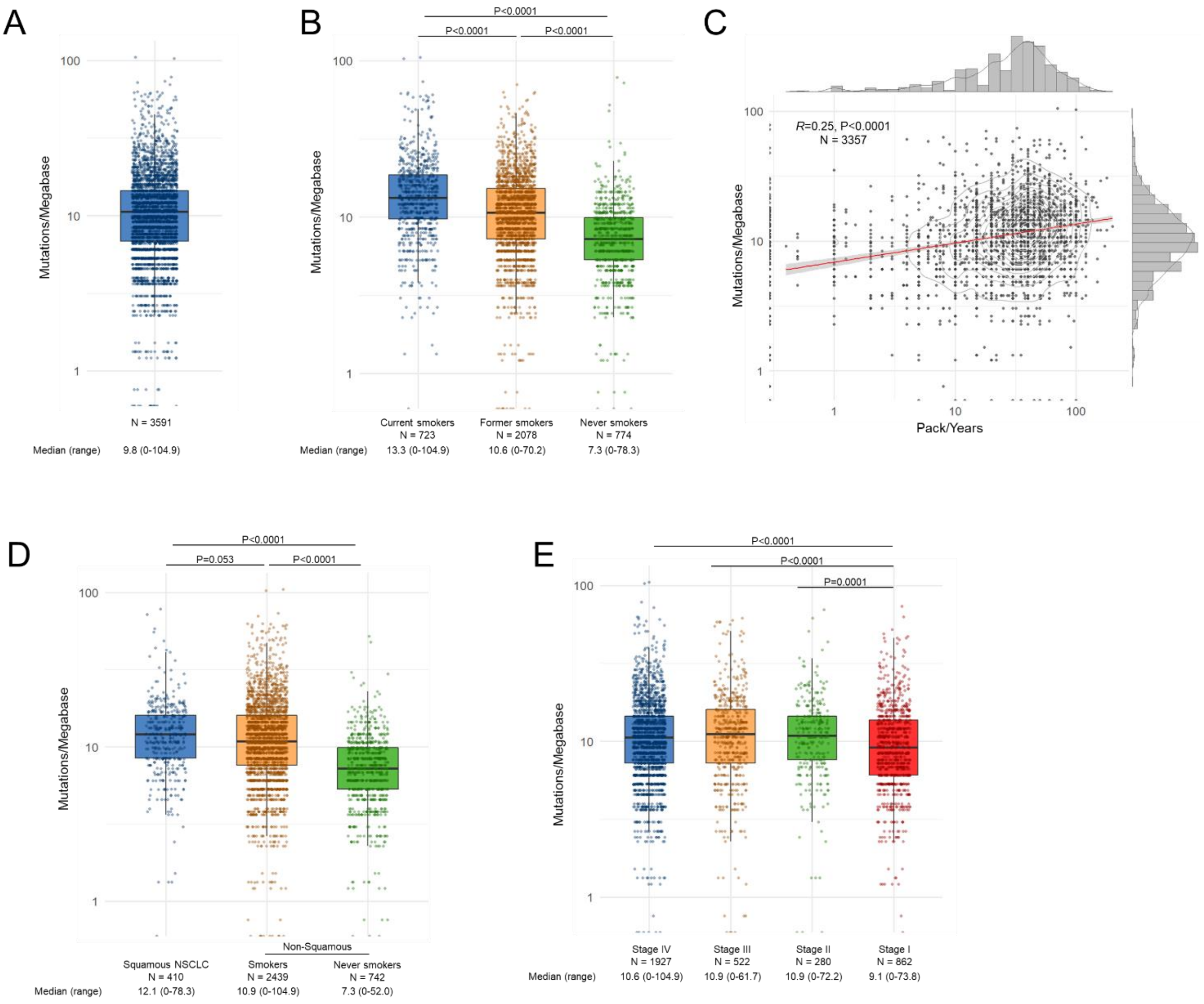

eFigure 2. (**A**) Tumor Mutational Burden of 3591 NSCLCs Which Underwent Next-Generation Sequencing at the Dana-Farber Cancer Institute, Correlation Between TMB With (**B**) tobacco History, (**C**) Number of Tobacco Pack-Years, (**D**) Tumor Histology, and (**E**) NSCLC Stage at the Time of Next-Generation Sequencing.

© 2022 Ricciuti B et al. *JAMA Oncology.*

eFigure 3

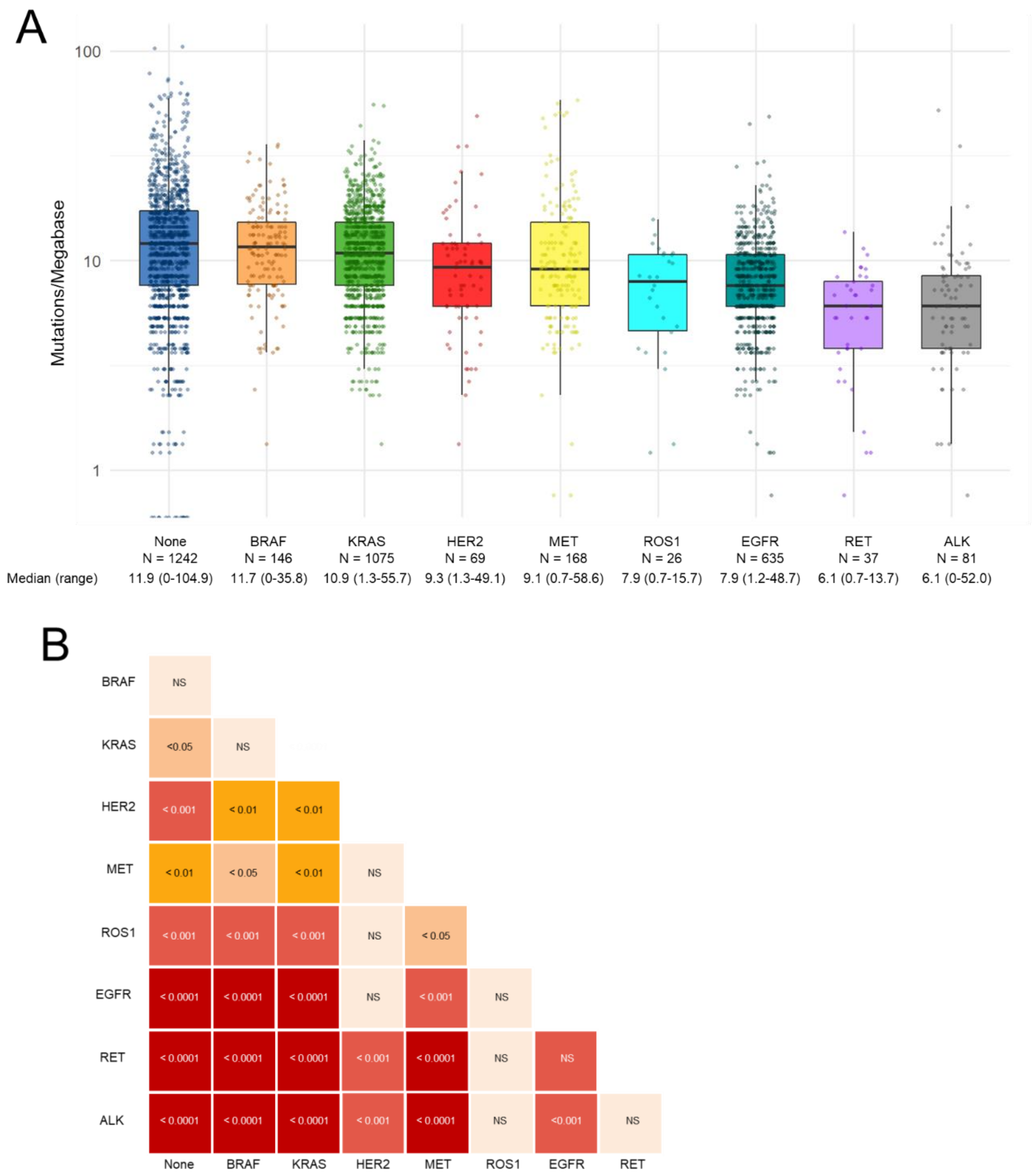

eFigure 3. (**A**) TMB Distributions According to NSCLC Genotype and (**B**) Q Values for Pairwise Comparisons of Tumor Genotype in Comparison to One Another (Benjamini-Hochberg Procedure). Samples with concurrent mutations in ≥2 driver mutations were excluded from these panels.

© 2022 Ricciuti B et al. *JAMA Oncology.*

eFigure 4

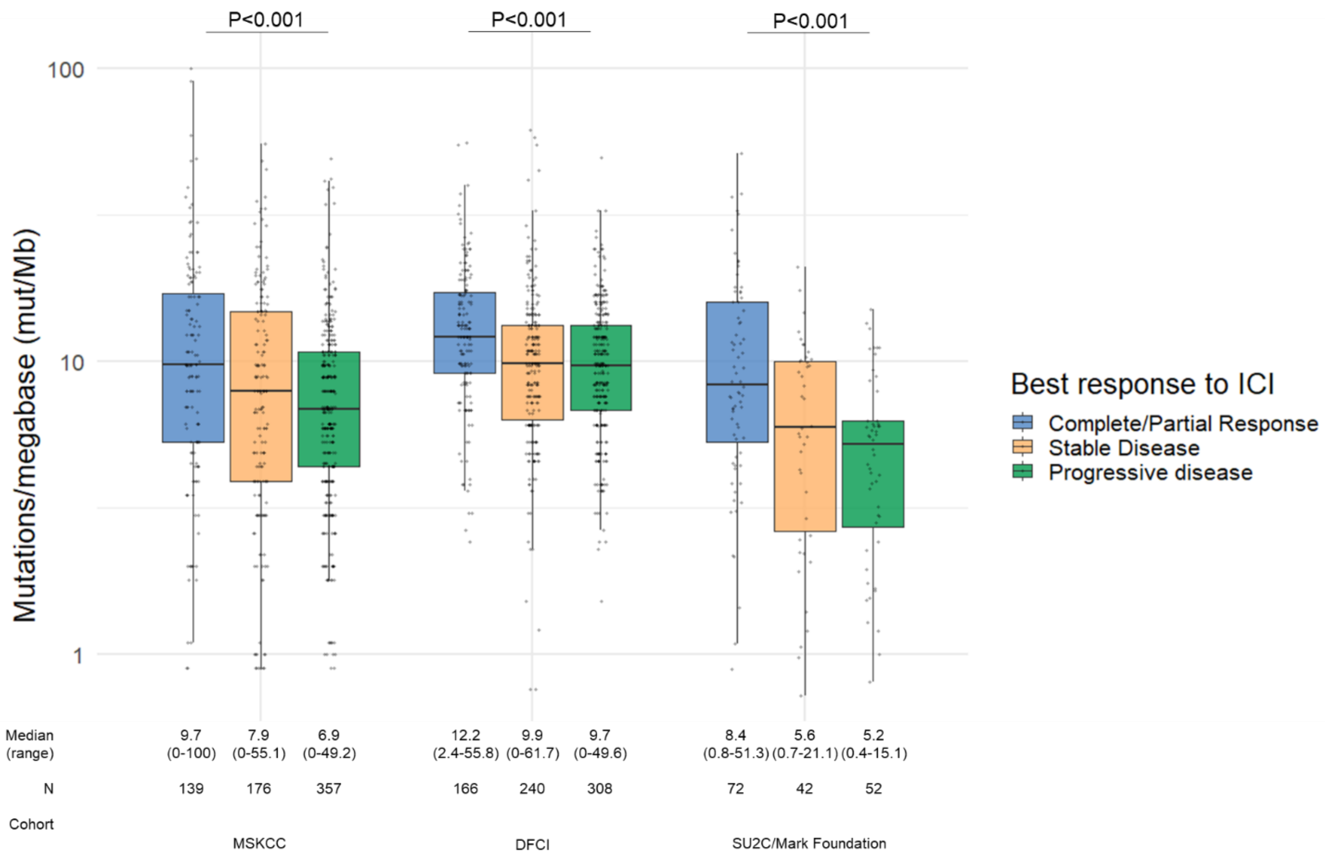

eFigure 4. Box Plot Showing the Distribution of TMB Among Patients With Non-Small Cell Lung Cancer Who Experienced a Complete/Partial Response, Stable Disease, and Progressive Disease as Best Response to PD-(L)1 Inhibition in the MSKCC, DFCI, and SU2C/Mark Foundation Cohorts.

© 2022 Ricciuti B et al. *JAMA Oncology.*

eFigure 5

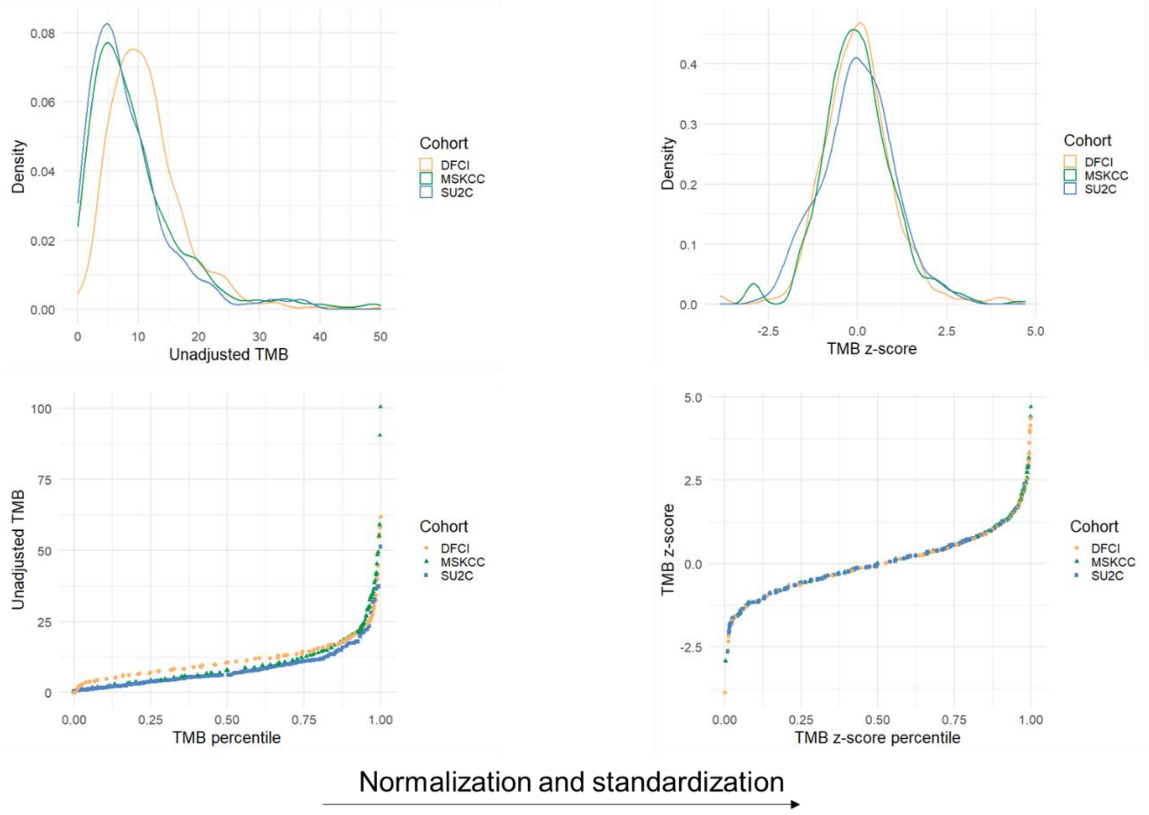

eFigure 5. Normalization and Standardization of TMB Distributions Bring the Next-Generation Sequencing (MSK-IMPACT and DFCI OncoPanel) and WES Cohort (SU2C/Mark Foundation) Distributions Into Alignment. The left side shows the kernel density plot of unadjusted TMB values in each cohort, and the right side shows the transformed density plot of TMB z-scores that demonstrate high overlap.

© 2022 Ricciuti B et al. *JAMA Oncology.*

eFigure 6

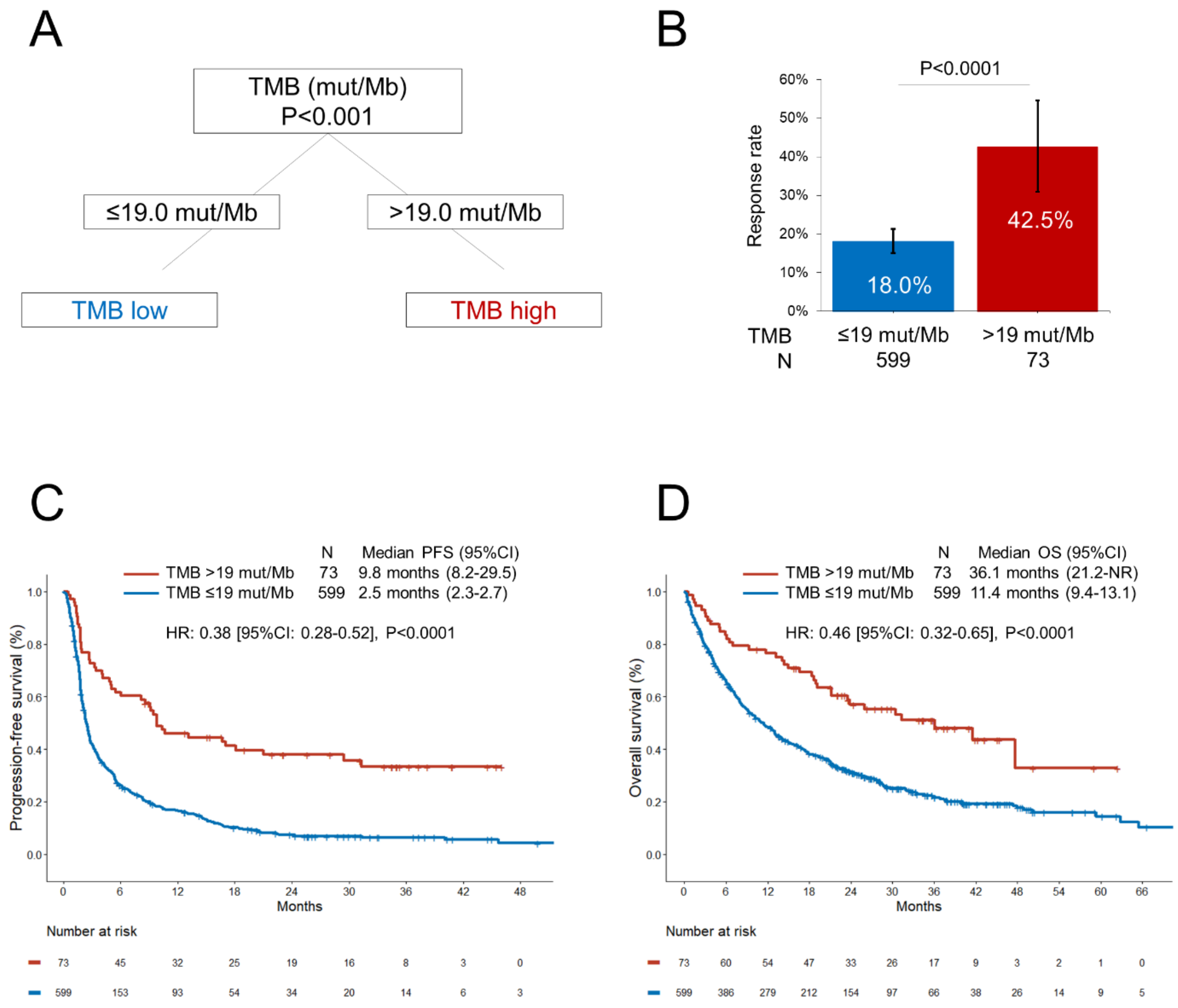

eFigure 6. (**A**) Unbiased Regression Tree Modeling the Objective Response to PD-(L)1 Blockade as Function of Tumor Mutational Burden Identified an Optimal Threshold of 19.0 Mutations/Megabase That Discriminates Responders Versus Nonresponders in the MSKCC Discovery Cohort, (**B**) Objective Response Rate, (**C**) Progression-Free, and (**D**) Overall Survival to Immunotherapy in Patients With TMB-High (>19 mut/Mb) vs TMB-Low (≤19 mut/Mb) NSCLC in the MSKCC Discovery Cohort.

© 2022 Ricciuti B et al. *JAMA Oncology.*

eFigure 7

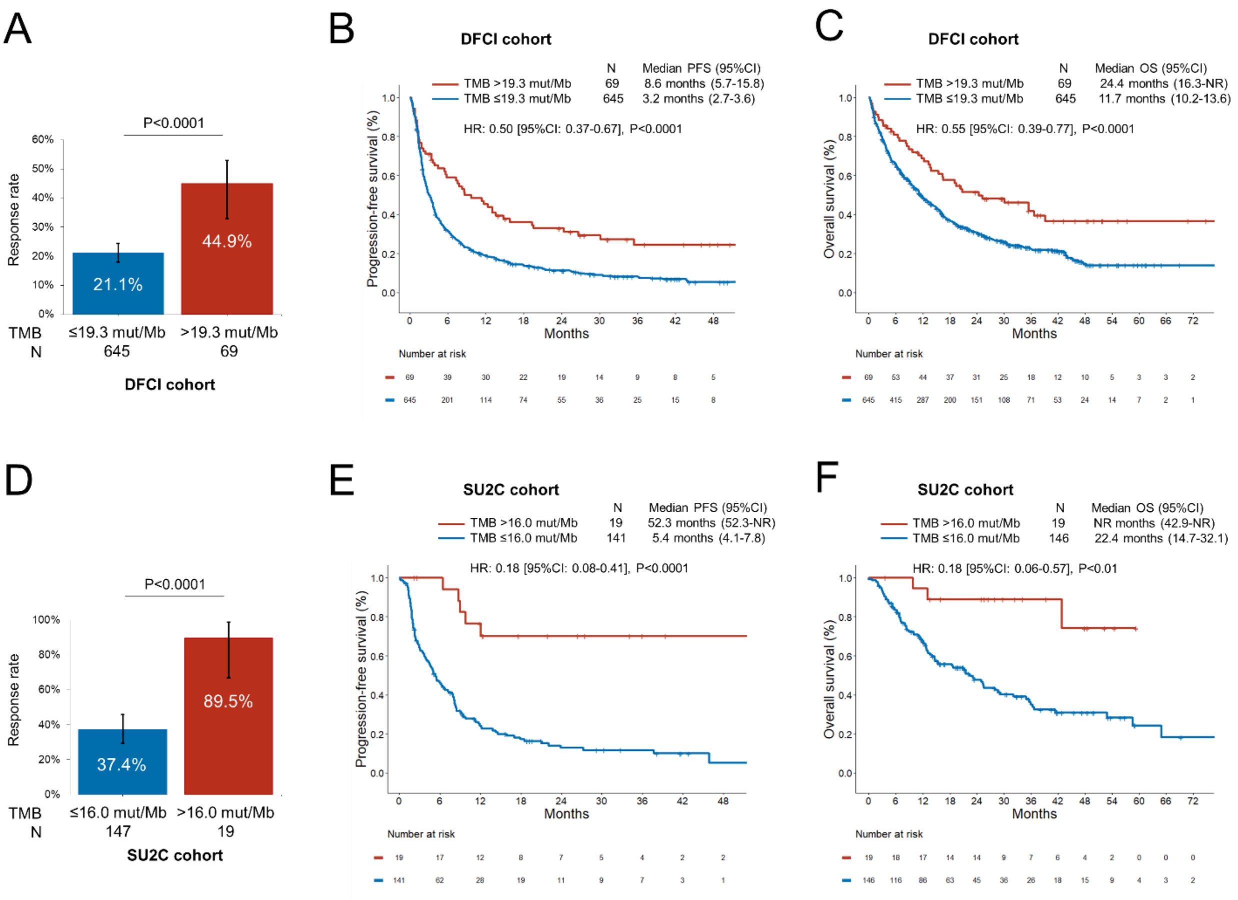

eFigure 7. (**A**) Objective Response Rate, (**B**) Progression-Free, and (**C**) Overall Survival to Immunotherapy in the DFCI Cohort According to TMB-High (>19.3 mut/Mb/TMB Z-Score >1.16) Versus TMB-Low TMB (≤19.3 mut/Mb/TMB Z-Score ≤1.16), (**D**) Objective Response Rate, (**E**) Progression-Free, and (**F**) Overall Survival to Immunotherapy According to TMB-High (>16.0 mut/Mb/TMB Z-Score >1.16) Versus TMB-Low TMB (≤16.0 mut/Mb/TMB Z-Score ≤1.16) Versus Low Harmonized TMB.

© 2022 Ricciuti B et al. *JAMA Oncology.*

eFigure 8

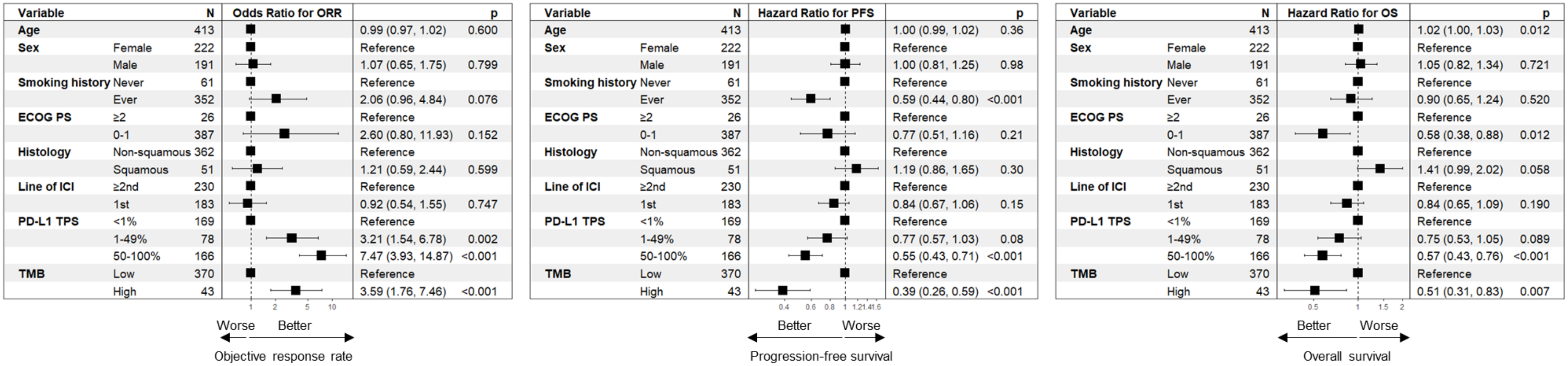

eFigure 8. Multivariable Analysis for Response, Progression-Free, and Overall Survival to PD-(L)1 Blockade in the Memorial Sloan Kettering Cancer Center Cohort.

© 2022 Ricciuti B et al. *JAMA Oncology.*

eFigure 9

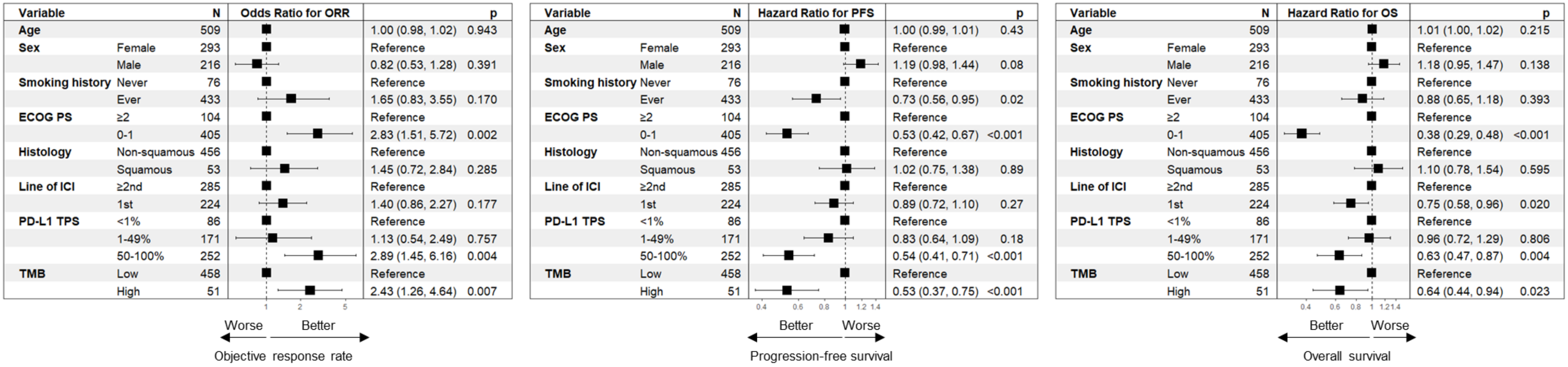

eFigure 9. Multivariable Analysis for Response, Progression-Free, and Overall Survival to PD-(L)1 Blockade in the Dana-Farber Cancer Institute Cohort.

© 2022 Ricciuti B et al. *JAMA Oncology.*

eFigure 10

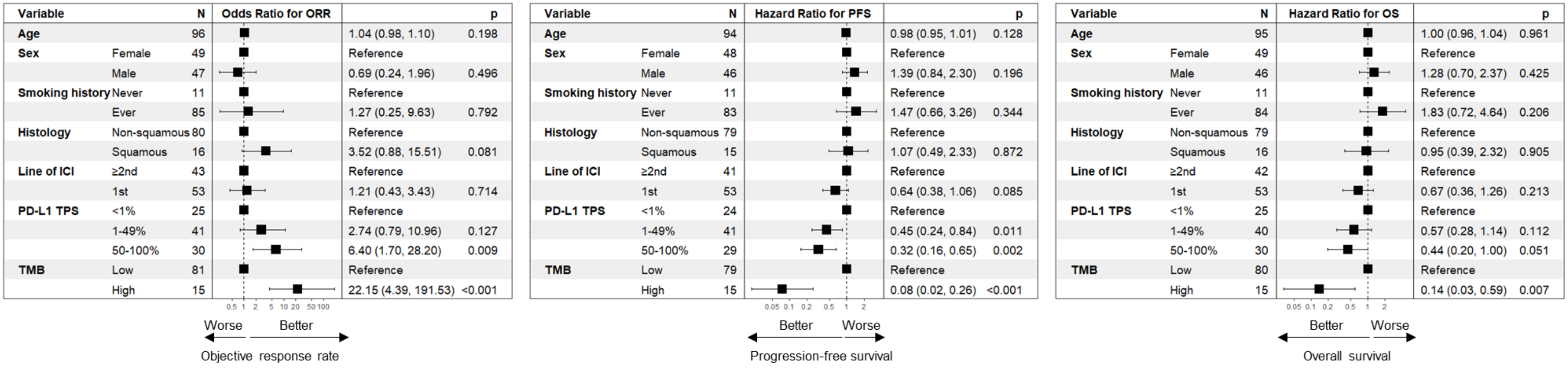

eFigure 10. Multivariable Analysis for Response, Progression-Free, and Overall Survival to PD-(L)1 Blockade in the Stand Up 2 Cancer/Mark Foundation Cohort.

© 2022 Ricciuti B et al. *JAMA Oncology.*

eFigure 11.

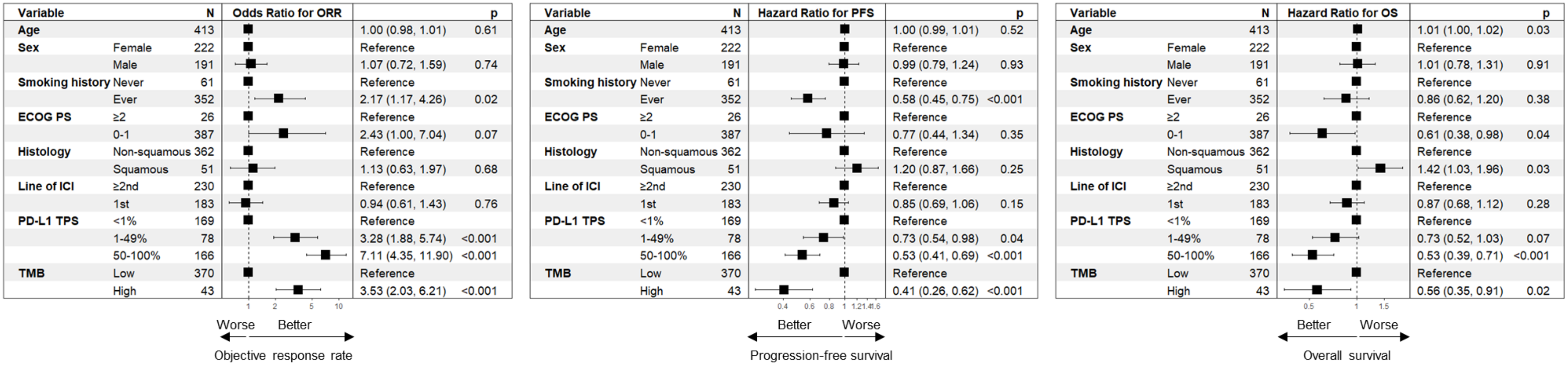

eFigure 11. Multivariable Analysis With Inverse Probability Weighting for PD-L1 Expression for Response, Progression-Free, and Overall Survival to PD-(L)1 Blockade in the Memorial Sloan Kettering Cancer Center Cohort.

© 2022 Ricciuti B et al. *JAMA Oncology.*

eFigure 12.

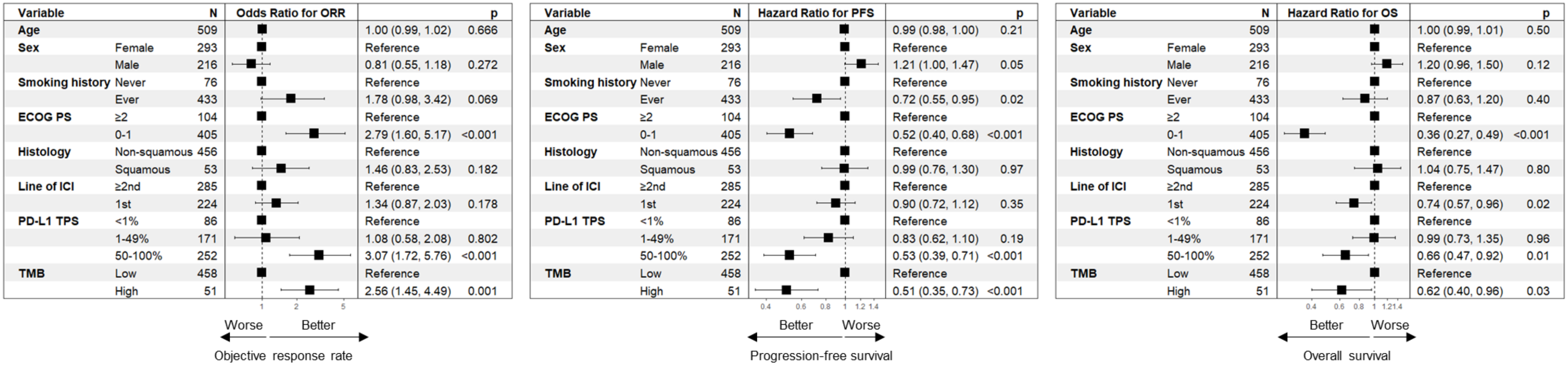

eFigure 12. Multivariable Analysis With Inverse Probability Weighting for PD-L1 Expression for Response, Progression-Free, and Overall Survival to PD-(L)1 Blockade in the Dana-Farber Cancer Institute Cohort.

© 2022 Ricciuti B et al. *JAMA Oncology.*

eFigure 13.

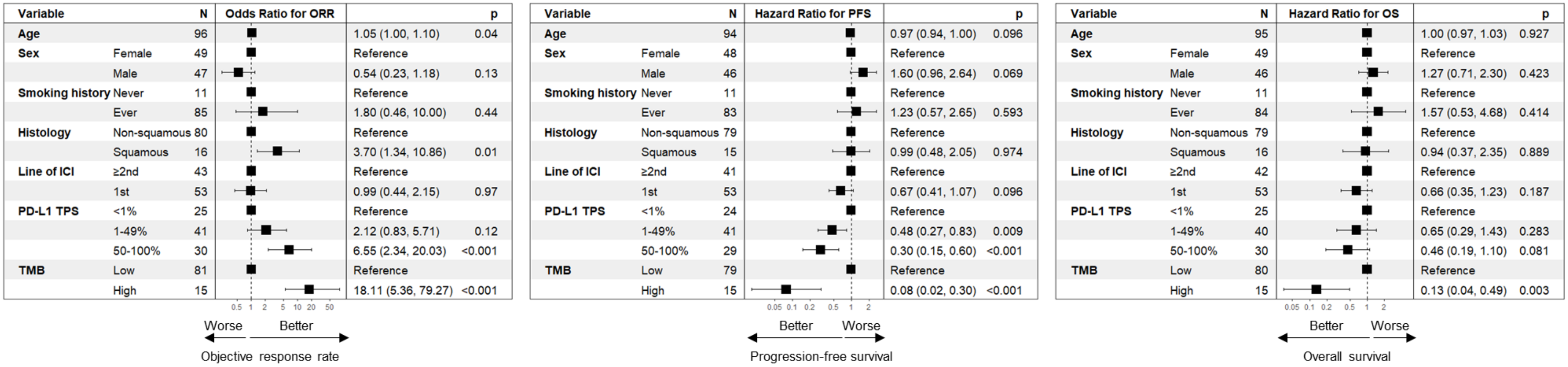

eFigure 13. Multivariable Analysis With Inverse Probability Weighting for PD-L1 Expression for Response, Progression-Free, and Overall Survival to PD-(L)1 Blockade in the Stand Up 2 Cancer/Mark Foundation Cohort.

© 2022 Ricciuti B et al. *JAMA Oncology.*

eFigure 14

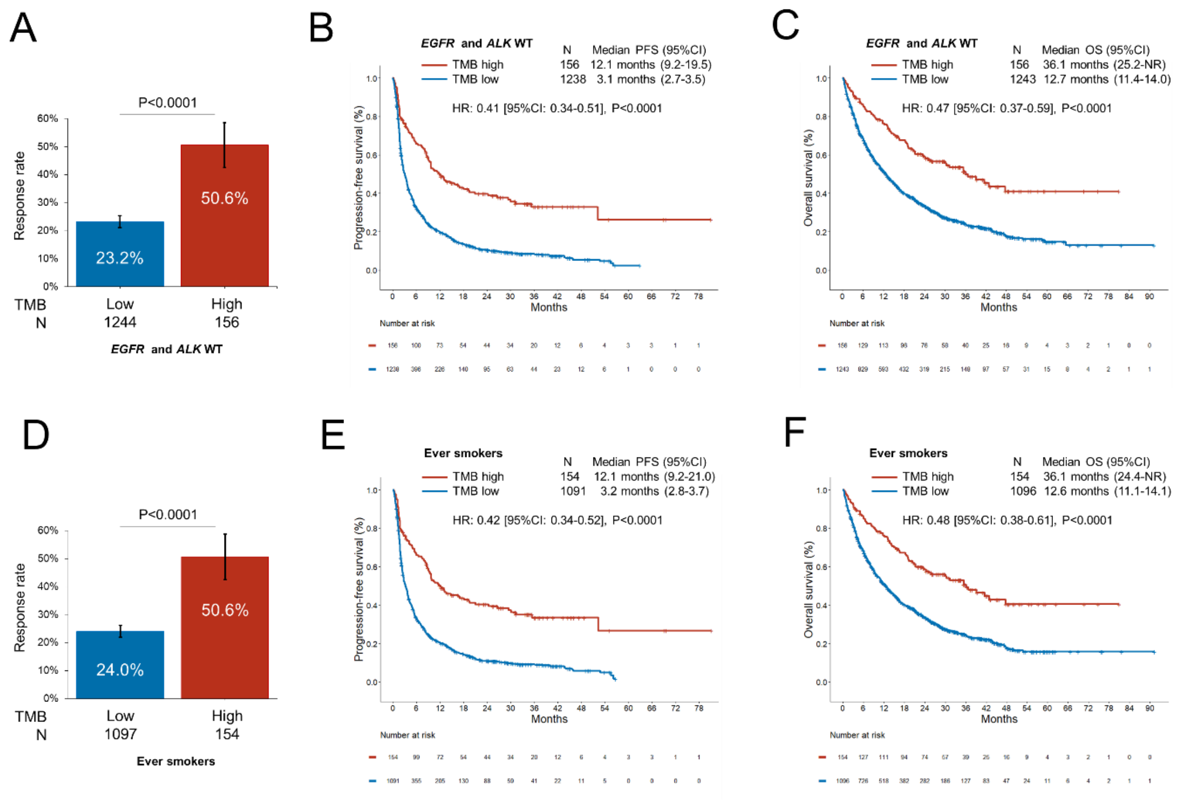

eFigure 14. (**A**) Objective Response Rate, (**B**) Progression-Free Survival, and (**C**) Overall Survival in Patients With High Versus Low Harmonized TMB in the Pooled Cohort of NSCLCs Treated With PD-(L)1 Blockade From DFCI, MSKCC, and the SU2C/Mark Foundation Dataset, After Excluding *EGFR* and *ALK* Positive Cases. (**D**) Objective response rate, (**E**) progression-free survival, and (**F**) overall survival in patients with high versus low harmonized TMB in the pooled cohort of NSCLCs treated with PD-(L)1 blockade from DFCI, MSKCC, and the SU2C/Mark Foundation dataset, after excluding never smokers. WT, wild type.

© 2022 Ricciuti B et al. *JAMA Oncology.*

eFigure 15

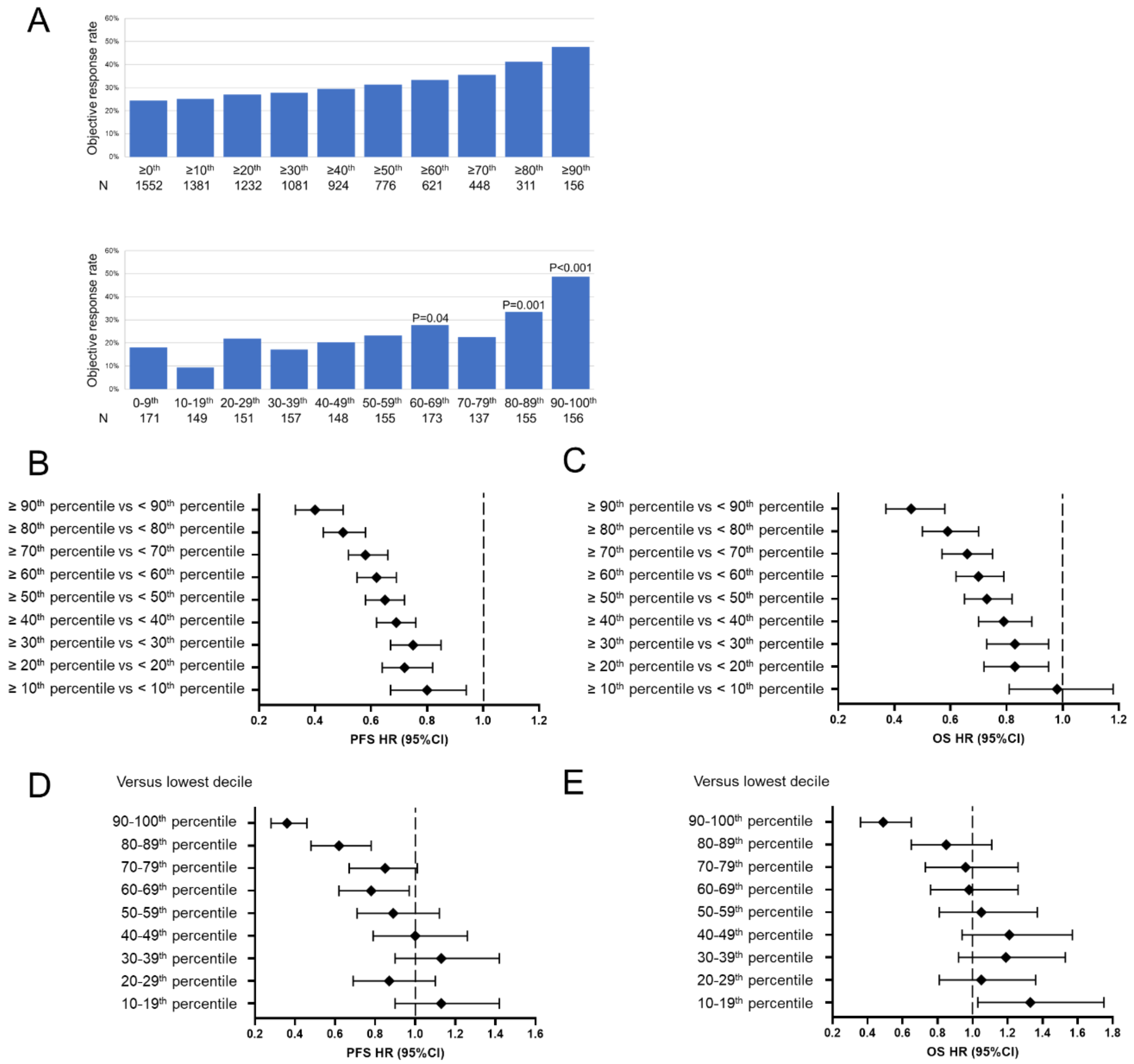

eFigure 15. (**A**) Objective Response Rate by Increasing TMB Percentiles Thresholds (Upper Panel), and in Each TMB Decile (Lower Panel) in the Combined Cohort. P values are comparing each decile with the lowest decile of TMB (0-9th). (**B**) Forest plot for progression-free and (**C**) overall survival to PD-(L)1 blockade according to increasing TMB thresholds in the pooled cohort (MSKCC + DFCI + SU2C, N = 1552). (**D**) Forest plot for progression-free and (**E**) overall survival to PD-(L)1 blockade in each TMB decile versus the lowest decile, as reference, in the pooled cohort (MSKCC + DFCI + SU2C, N = 1552).

© 2022 Ricciuti B et al. *JAMA Oncology.*

eFigure 16

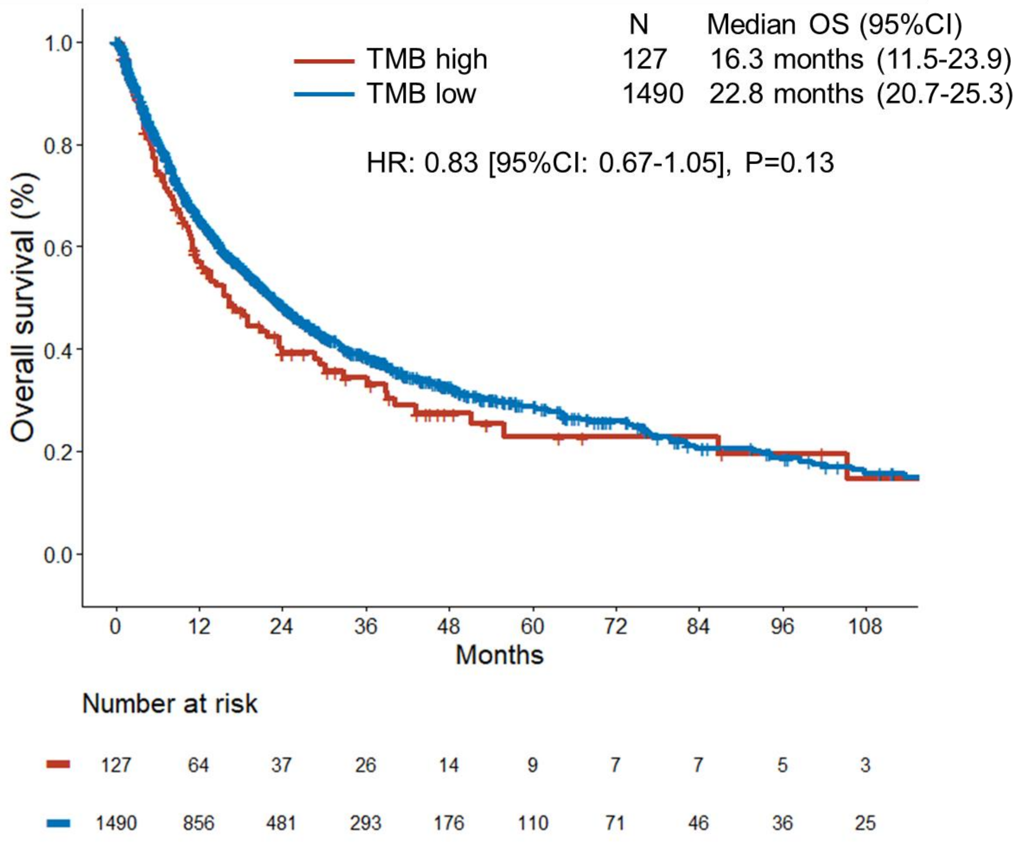

eFigure 16. Overall Survival in Patients at DFCI and MSKCC With Advanced Non-Small Cell Lung Cancer Who Never Received Immunotherapy According to TMB Levels.

© 2022 Ricciuti B et al. *JAMA Oncology.*

eFigure 17

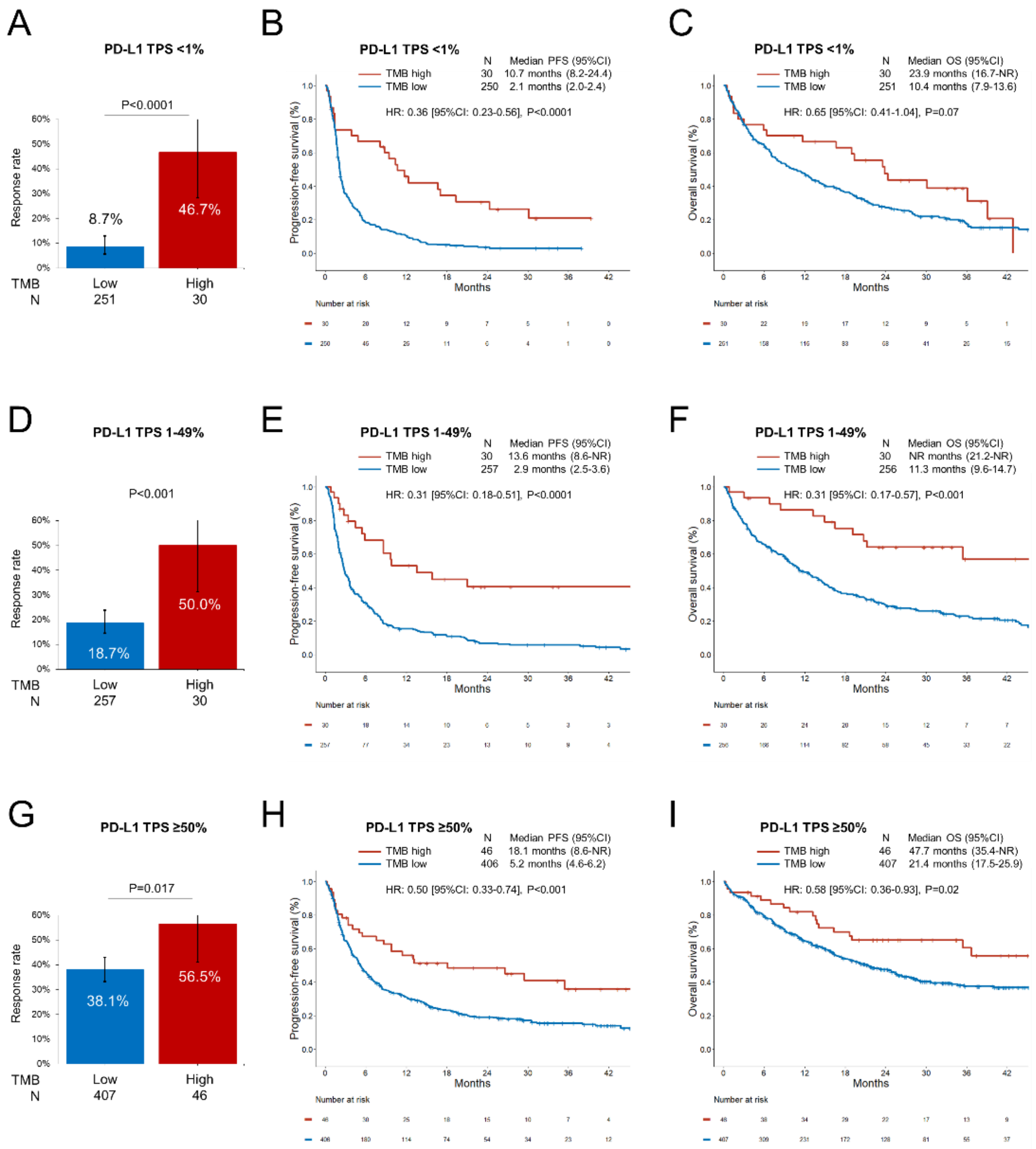

eFigure 17. (**A**) Response Rate, (**B**) Progression-Free, and (**C**) Overall Survival to PD-(L)1 Inhibition According to TMB Levels Among Non-Small Cell Lung Cancers With a PD-L1 TPS <1%. (**D**) Response rate, (**E**) progression-free, and (**F**) overall survival to PD-(L)1 inhibition according to TMB levels among non-small cell lung cancers with a PD-L1 TPS of 1-49%. (**G**) Response rate, (**H**) progression-free, and (**I**) overall survival to PD-(L)1 inhibition according to TMB levels among non-small cell lung cancers with a PD-L1 TPS ≥50%.

© 2022 Ricciuti B et al. *JAMA Oncology.*

eFigure 18

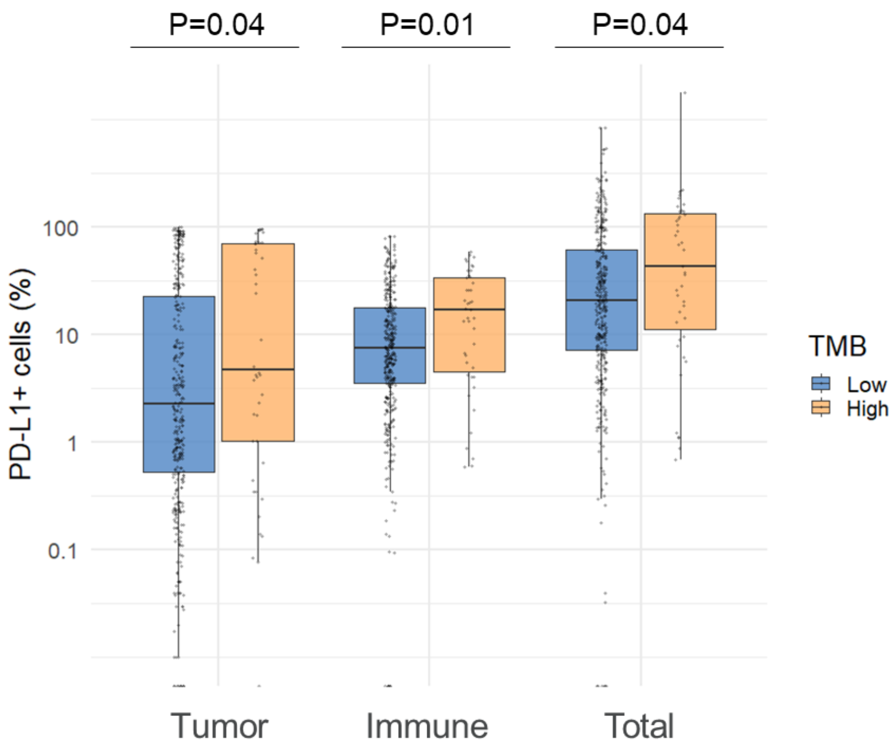

eFigure 18. Percentage of Tumor, Immune, and Total Cells With PD-L1 Expression Among NSCLC Samples With Low (N = 384) and High (N = 44) TMB Which Also Underwent Multiplexed Immunofluorescence at the DFCI.

© 2022 Ricciuti B et al. *JAMA Oncology.*

eFigure 19

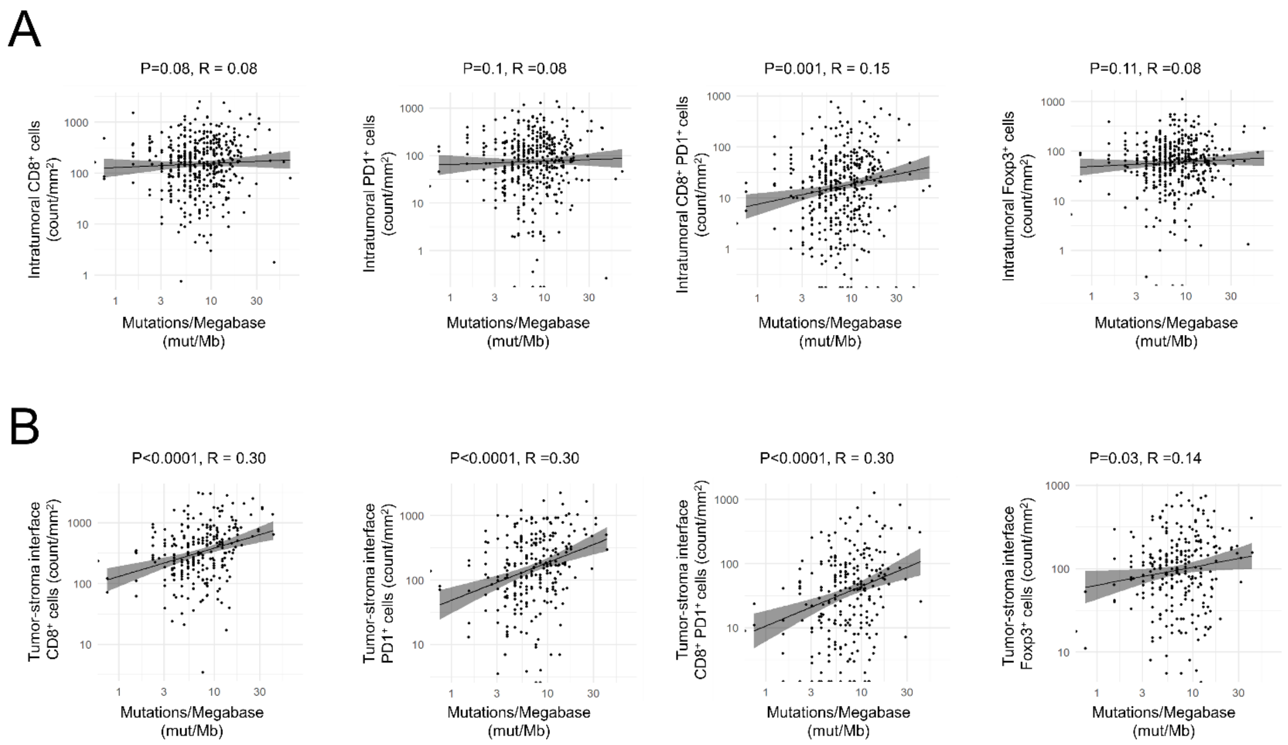

eFigure 19. Linear Correlation Between TMB and CD8$^+$, PD-1$^+$, CD8$^+$ PD-1$^+$, and Foxp3$^+$Cells Intratumorally (**A**) and at the Tumor-Stroma Interface (**B**) Among 428 NSCLCs at DFCI Which Underwent Multiplexed Immunofluorescence.

© 2022 Ricciuti B et al. *JAMA Oncology.*

eFigure 20

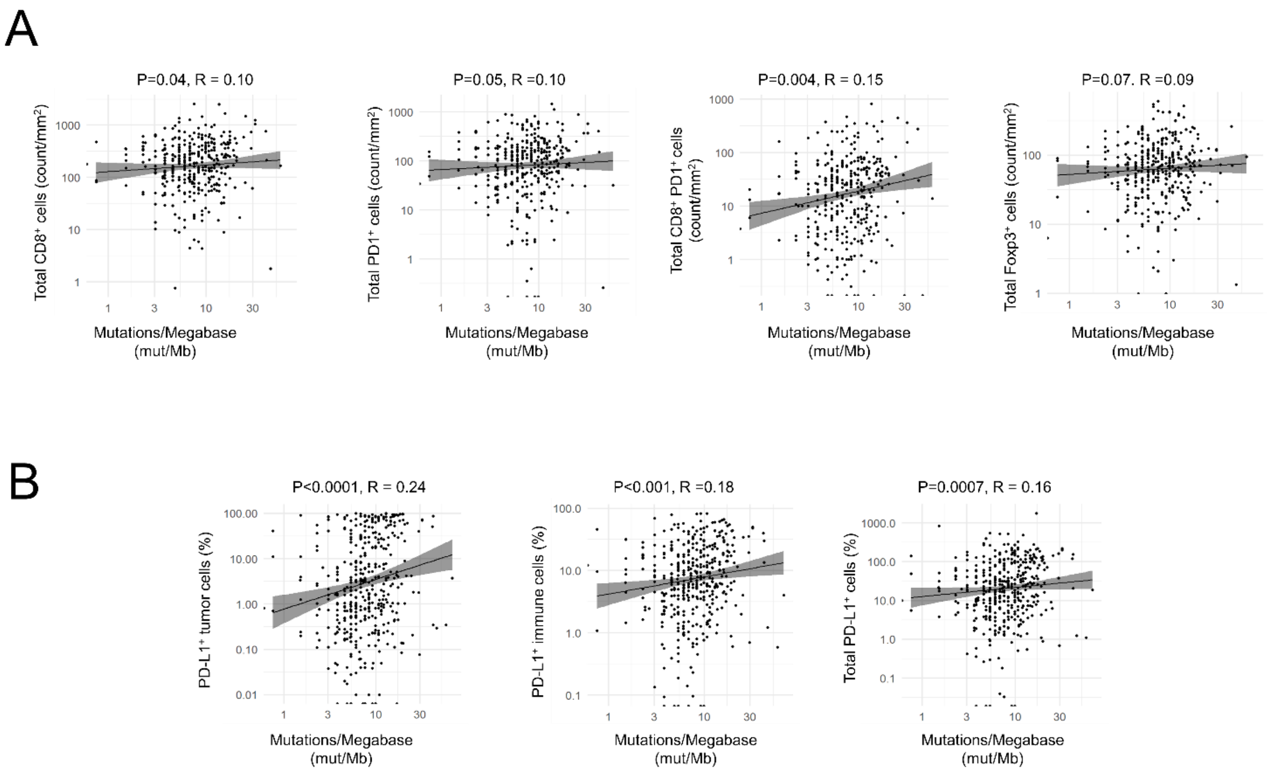

eFigure 20. Linear Correlation Between TMB and (**A**) Total $CD8^+$, $PD\text{-}1^+$, $CD8^+$ $PD\text{-}1^+$, and $Foxp3^+$ Cells, and (**B**) Linear Correlation Between Tumoral, Immune, and total PD-$L1^+$ Cells Among 428 NSCLCs at DFCI Which Underwent Multiplexed Immunofluorescence.

© 2022 Ricciuti B et al. *JAMA Oncology.*

eFigure 21

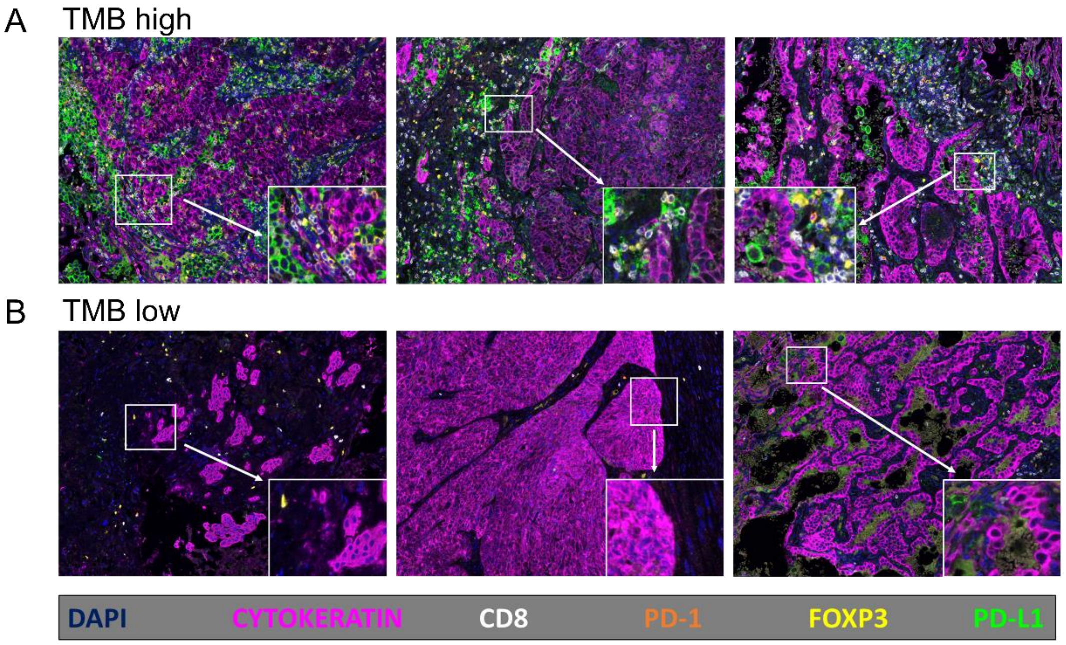

eFigure 21. Multiplexed Immunofluorescence for CD8, PD-1, Foxp3, PD-L1, in Three Index Cases With High TMB (**A**) and Three Index Cases With Low TMB (**B**).

© 2022 Ricciuti B et al. *JAMA Oncology.*

eFigure 22

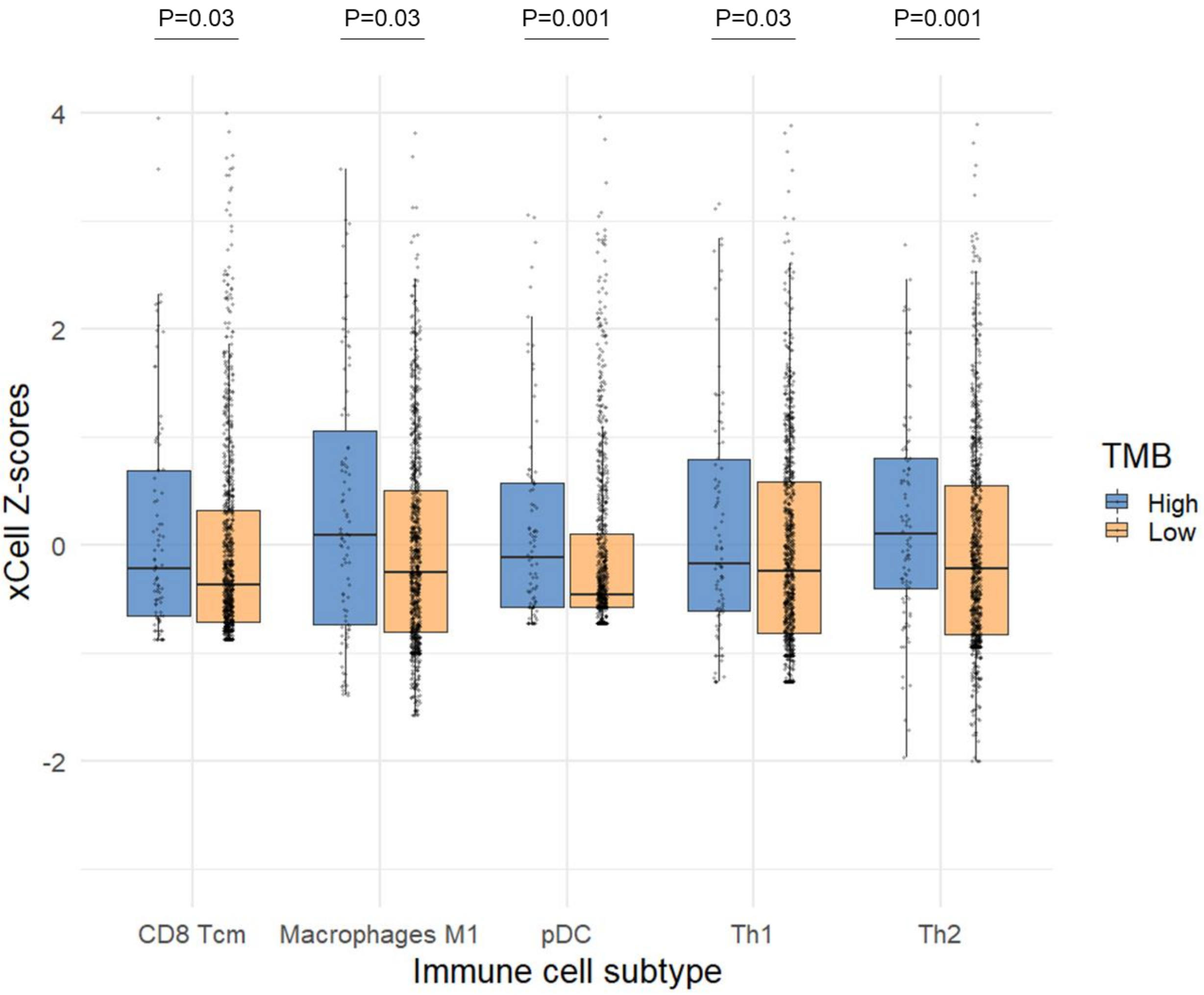

eFigure 22. Deconvolution of RNAseq Data From the NSCLC TCGA Dataset (N=998) Into Tumor-Associated Immune Cells, Showing Cell Types That Are Significantly Enriched in NSCLCs With High vs Low TMB.

© 2022 Ricciuti B et al. *JAMA Oncology.*

eFigure 23

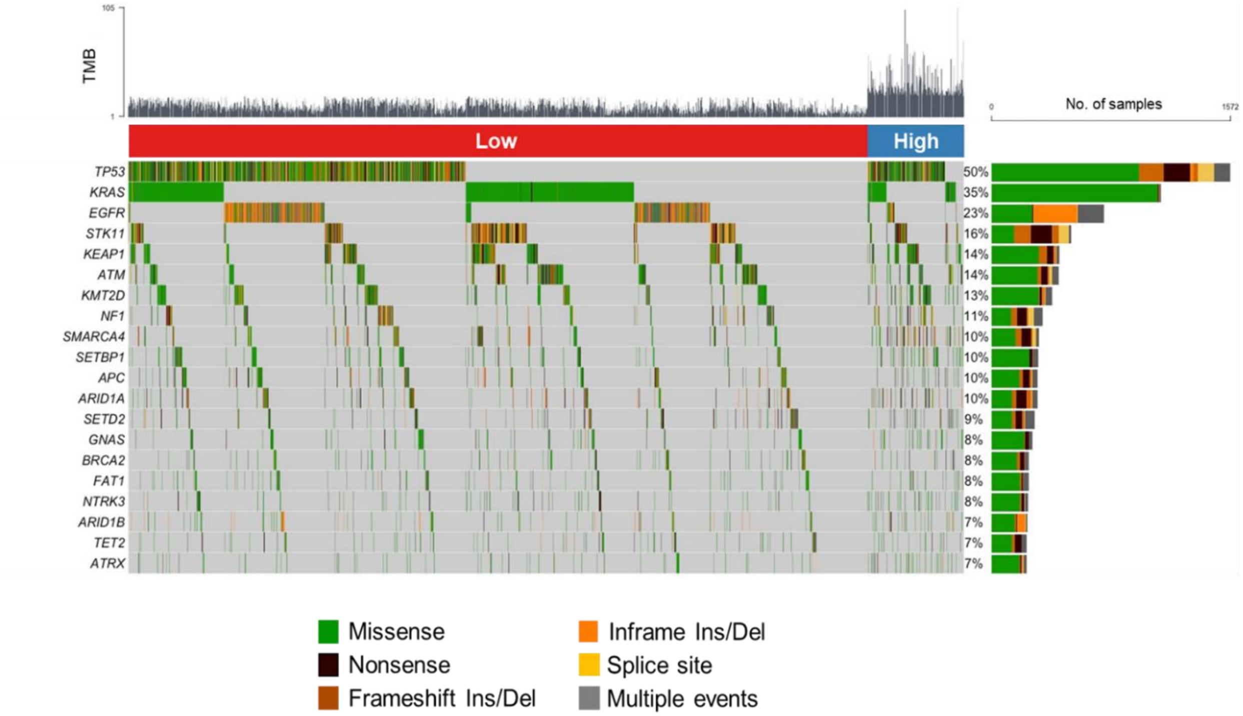

eFigure 23. OncoPrint Plot Showing the Top 20 Mutated Genes in 3168 Nonsquamous NSCLCs With High and Low TMB in the DFCI Genomic Cohort.

© 2022 Ricciuti B et al. *JAMA Oncology.*

eFigure 24

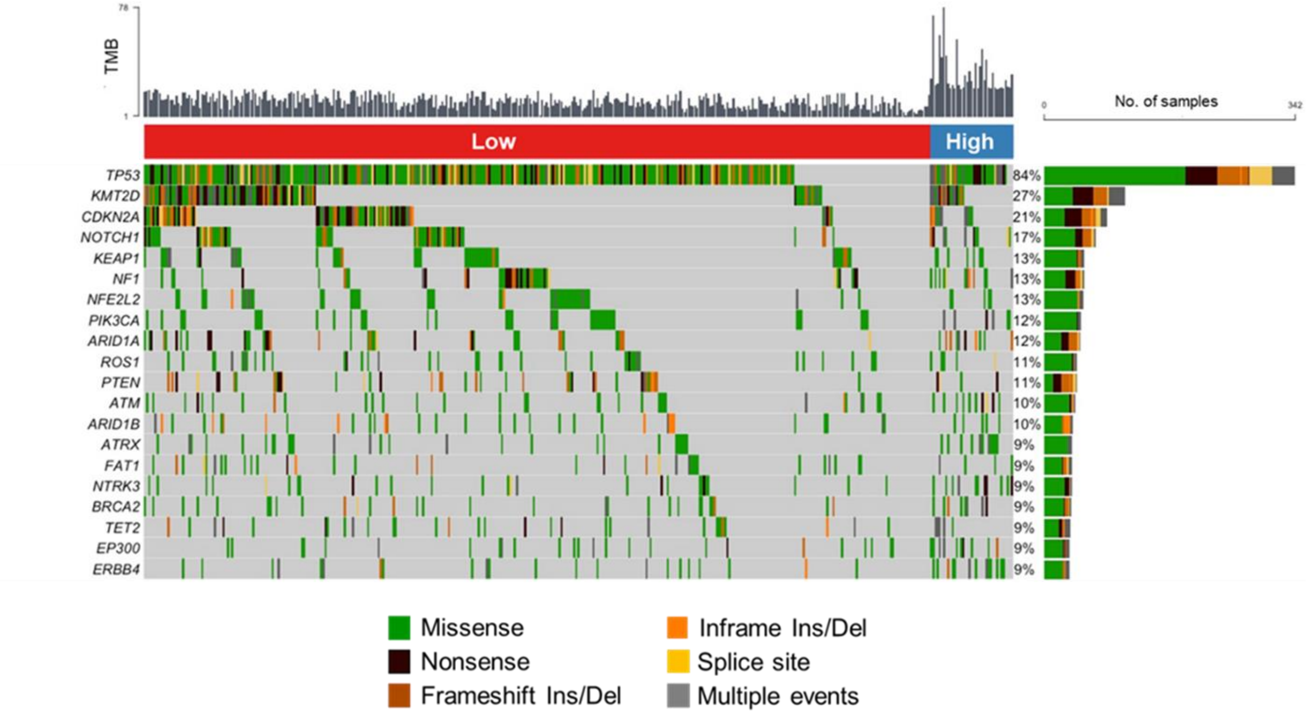

eFigure 24. OncoPrint Plot Showing the Top 20 Mutated Genes in 409 Squamous NSCLCs With High and Low TMB in the DFCI Genomic Cohort.

© 2022 Ricciuti B et al. *JAMA Oncology.*

eFigure 25

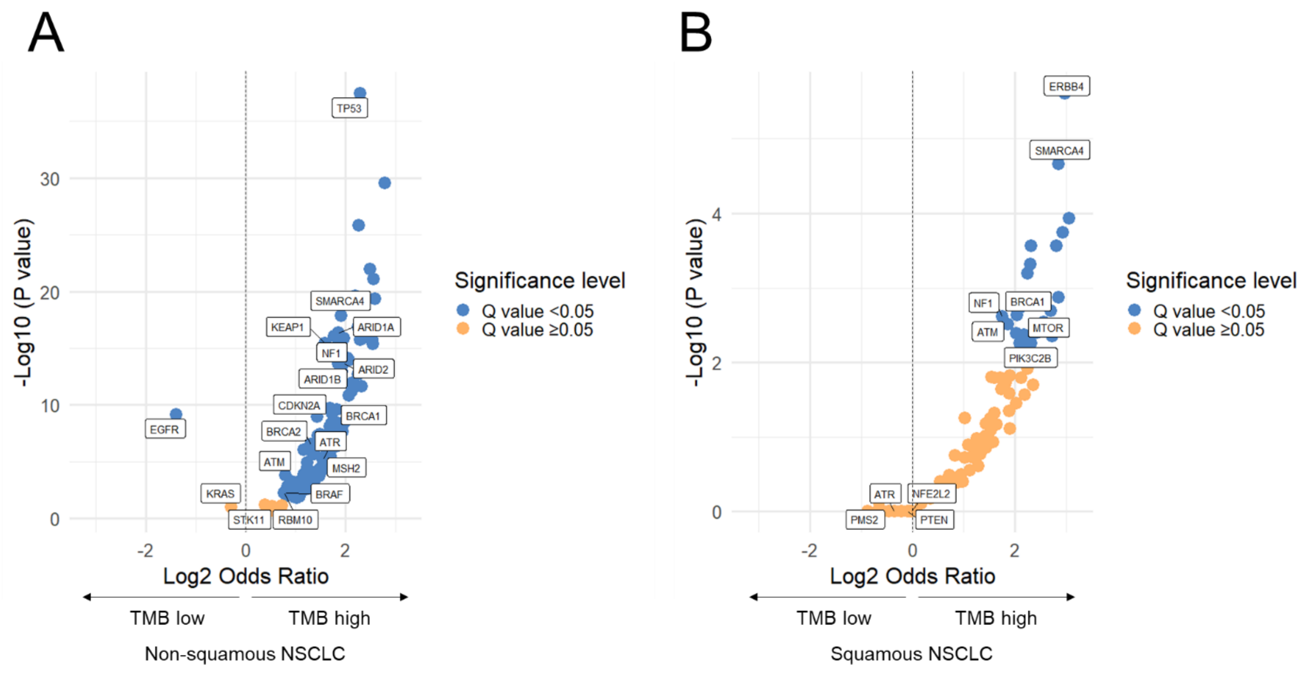

eFigure 25. Volcano Plot Showing Gene Mutations Enriched in TMB High Versus TMB Low (**A**) Nonsquamous (N=3168) and (**B**) Squamous (N=409) Non-Small Cell Lung Cancers in the DFCI Genomic Cohort.

© 2022 Ricciuti B et al. *JAMA Oncology.*

eFigure 26

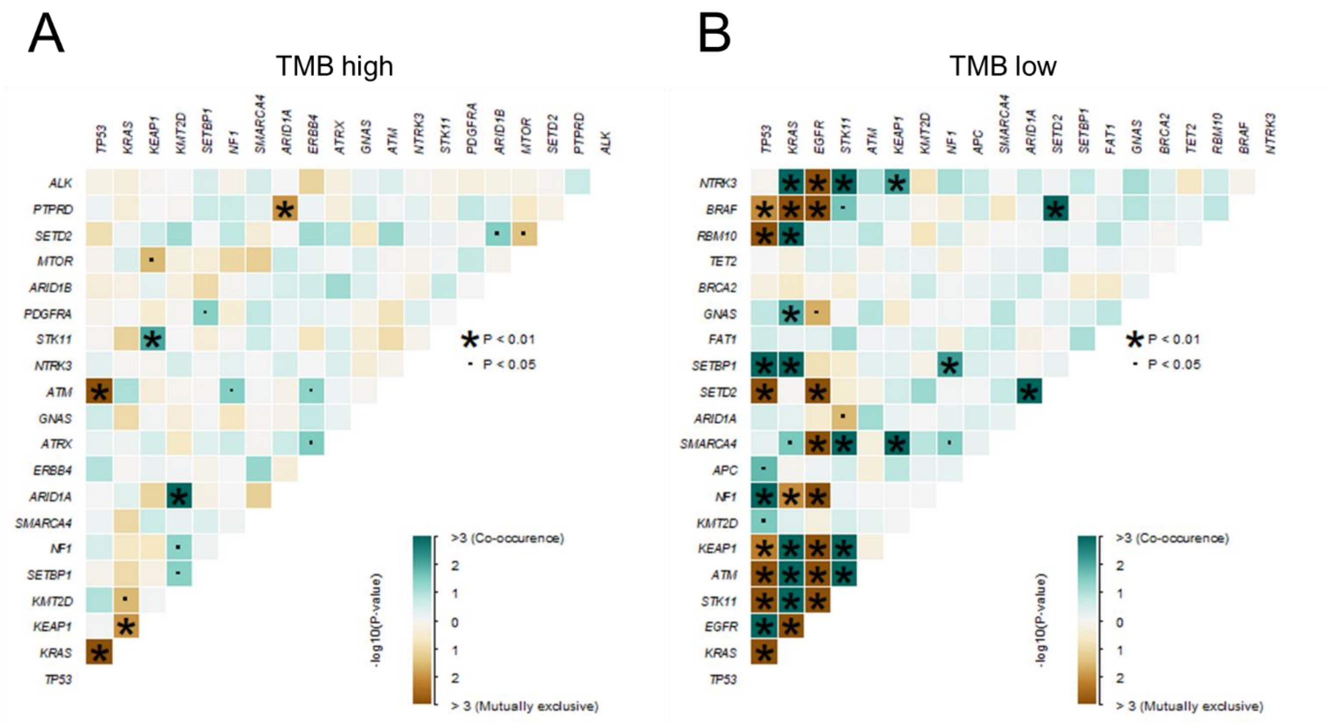

eFigure 26. Comutation Patterns Among Lung Non-Squamous Carcinomas With (**A**) High TMB (N=365) and (**B**) Low TMB (N=2803) in the DFCI Genomic Cohort. Five random samplings of 365 cases in the TMB low group confirmed significant co-occurrence of *KRAS/STK11* and *KRAS/KEAP1* mutation, indicating that the lack of co-mutation in these genes in the TMB high group is not influenced by the sample size.

© 2022 Ricciuti B et al. *JAMA Oncology.*

eFigure 27

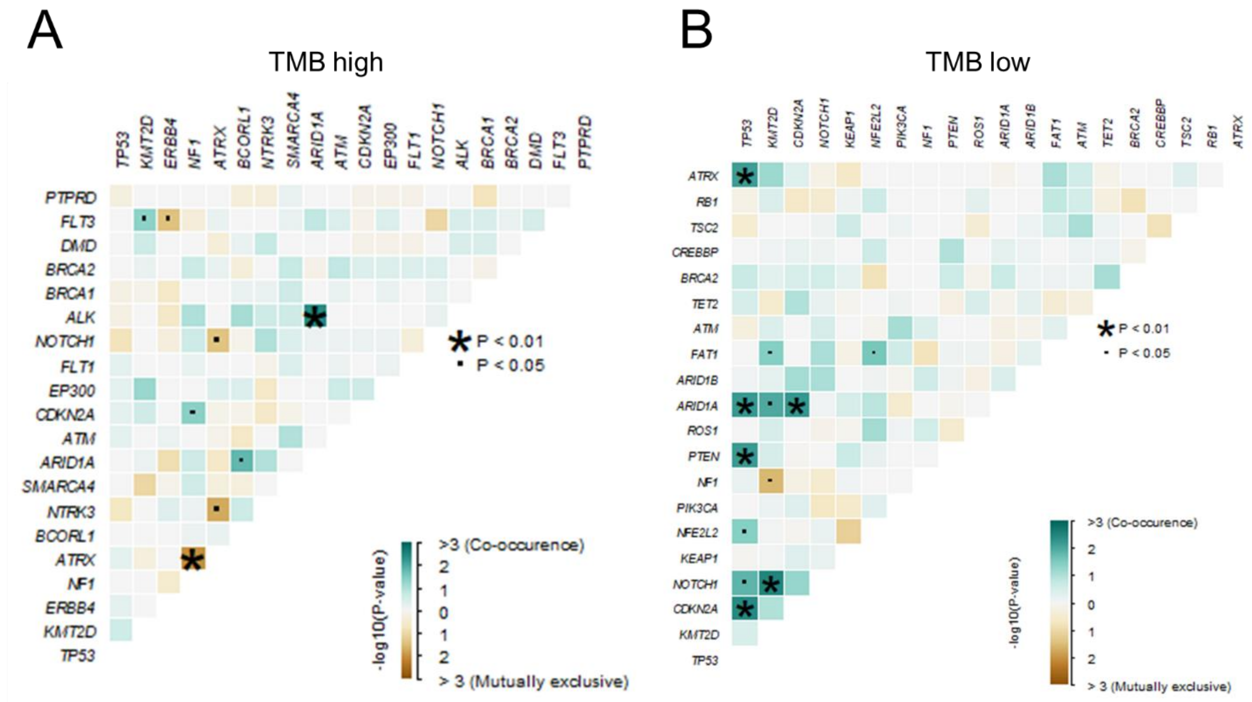

eFigure 27. Comutation Patterns Among Lung Squamous Carcinomas With (**A**) High TMB (N=39) and (**B**) Low TMB (N=370) in the DFCI Genomic Cohort.

© 2022 Ricciuti B et al. *JAMA Oncology.*

eFigure 28

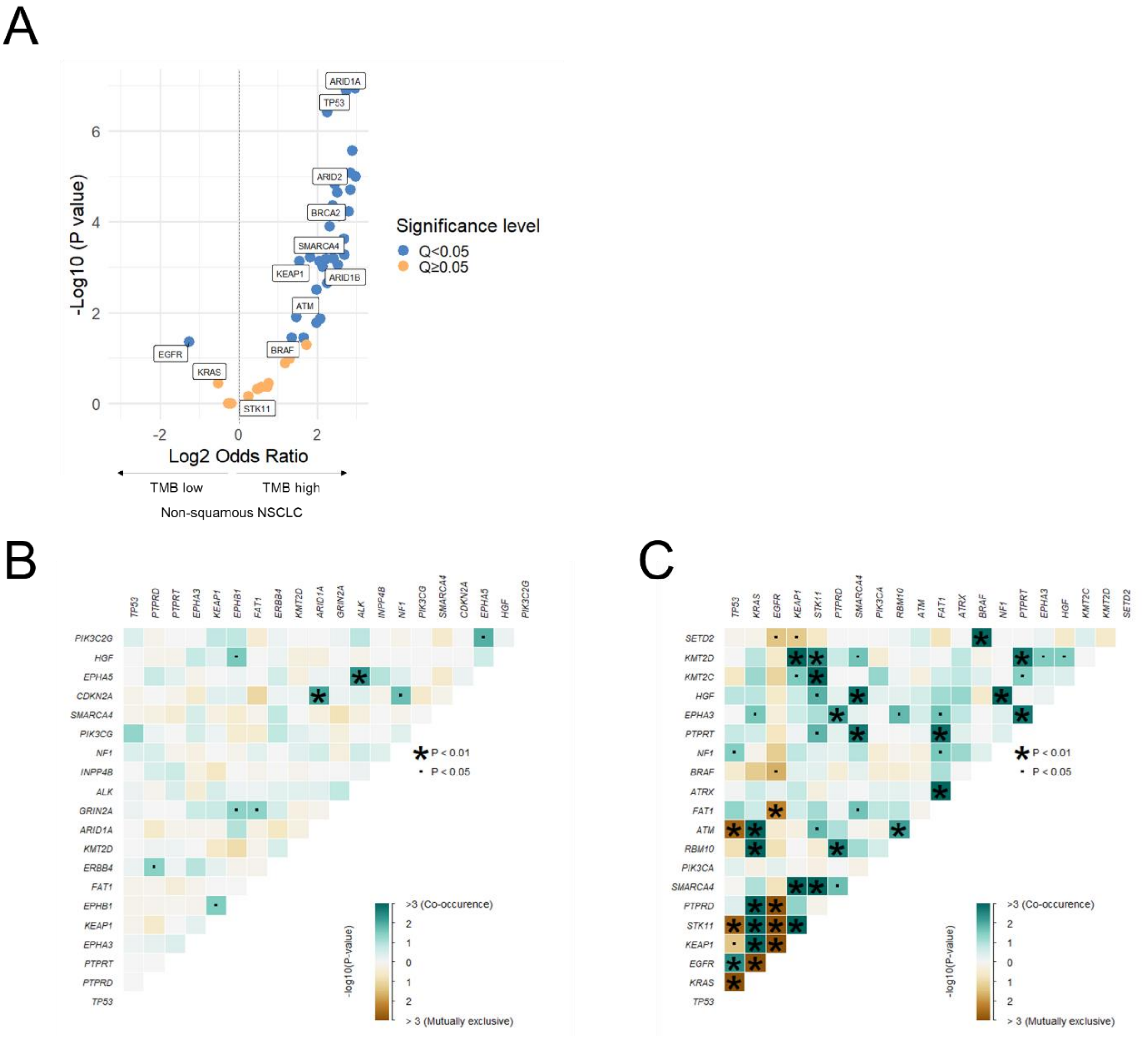

eFigure 28. (**A**) Volcano Plot Showing Gene Mutations Enriched in TMB High Versus TMB Low Nonsquamous NSCLC Among 915 Samples Which Underwent NGS at MSKCC. Co-mutation patterns among lung non-squamous carcinomas with (**B**) high TMB and (**C**) low TMB in the MSKCC genomic cohort.

© 2022 Ricciuti B et al. *JAMA Oncology.*

eFigure 29

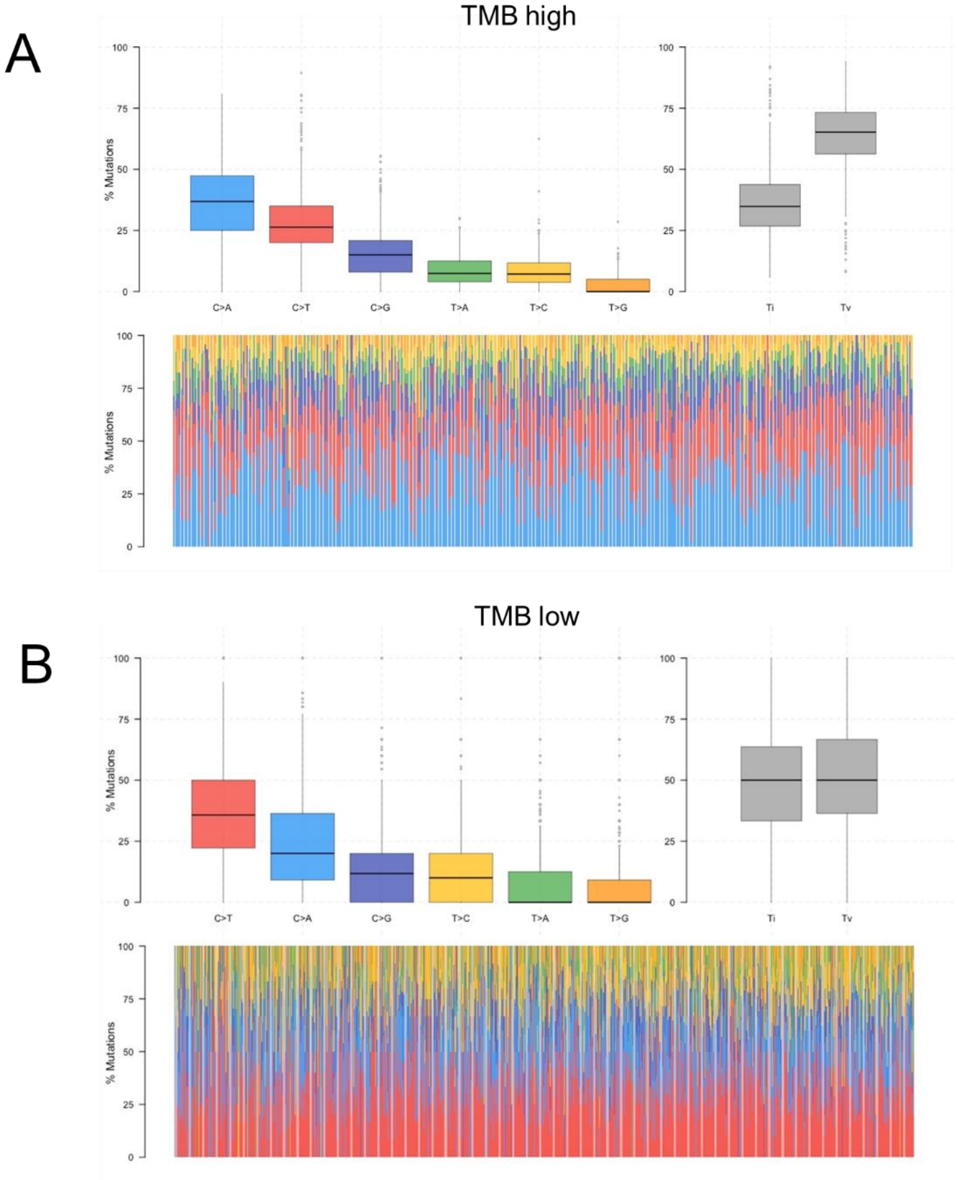

eFigure 29. (**A**) Boxplot Showing Overall Distribution of Nucleotide Conversions and Stacked Barplot Showing Fraction of Conversions in Each NSCLC Sample With TMB High in the DFCI Genomic Cohort (N=404) and (**B**) Boxplot Showing Overall Distribution of Nucleotide Conversions and Stacked Barplot Showing Fraction of Conversions in Each NSCLC Sample With TMB Low in the DFCI Genomic Cohort (N=3173).

© 2022 Ricciuti B et al. *JAMA Oncology.*

eFigure 30

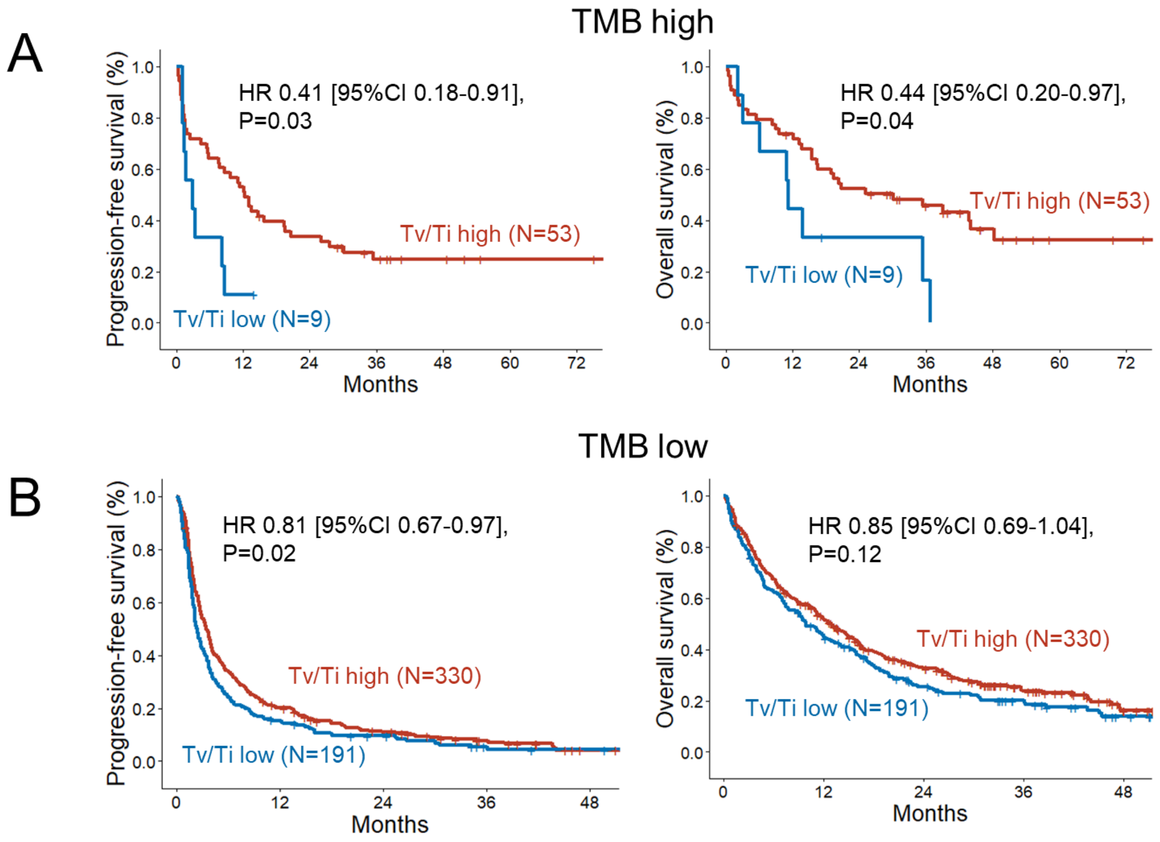

eFigure 30. (**A**) Progression-Free and Overall Survival to PD-(L)1 Blockade Among Patients With High (≥1) Versus Low (<1) Transversion/Transition Ratio Among Patients With TMB High and (**B**) Progression-Free and Overall Survival to PD-(L)1 Blockade Among Patients With High (≥1) Versus Low (<1) Transversion/Transition Ratio Among Patients With TMB Low.

© 2022 Ricciuti B et al. *JAMA Oncology.*

eFigure 31

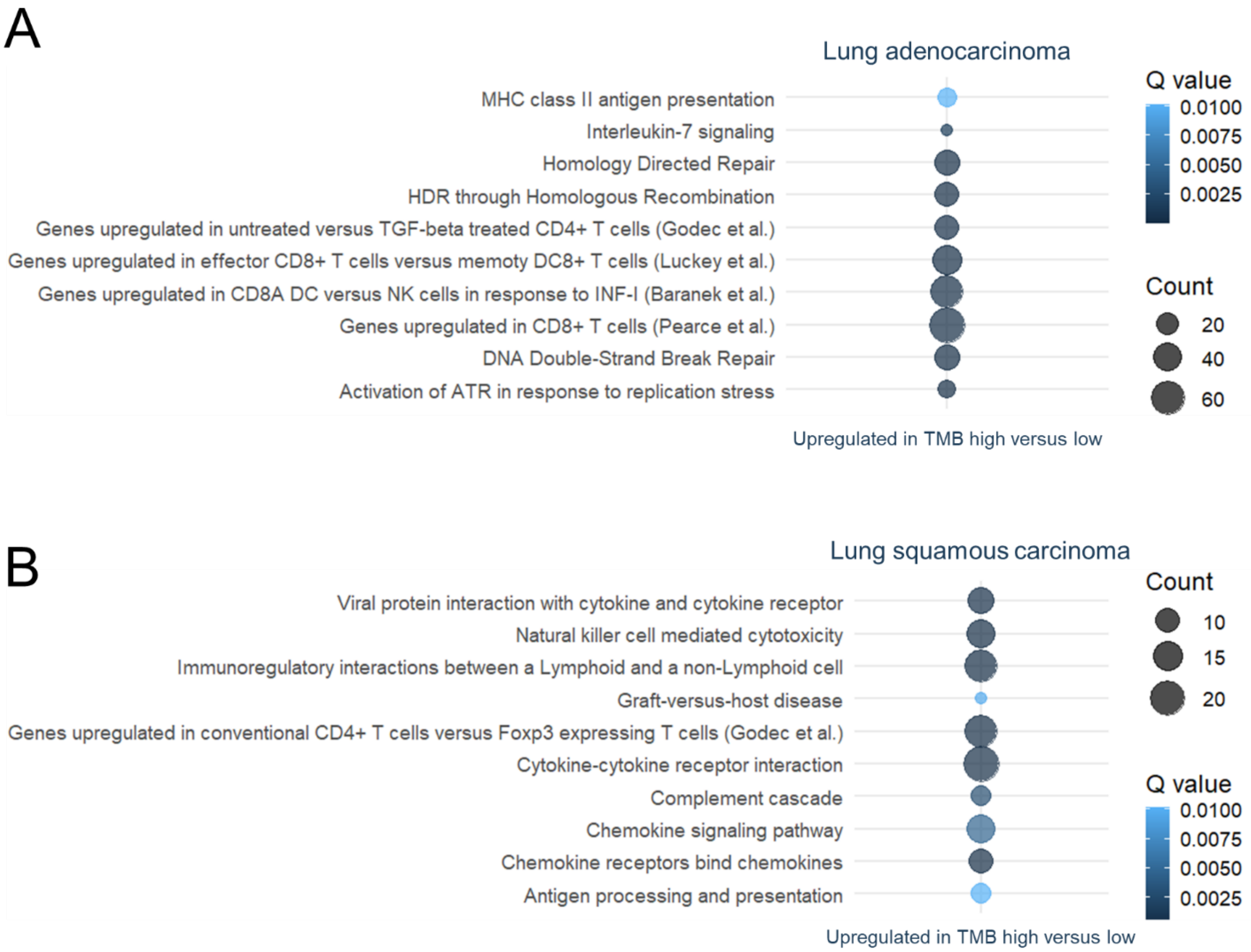

eFigure 31. Gene Set Enrichment Analysis Showing Prioritized Pathways Upregulated in TMB High Versus TMB Low NSCLC in (**A**) Lung Adenocarcinoma, and (**B**) Lung Squamous Carcinoma in the TCGA Cohort.

© 2022 Ricciuti B et al. *JAMA Oncology.*

eTable 1. Clinicopathologic and Genomic Characteristics of the 3591 NSCLCs Which Underwent Next-Generation Sequencing at the Dana-Farber Cancer Institute.

Clinical Characteristic	N = 3591 (%)
**Age, median (range)**	66 (18-99)
**Sex**	
Female	2111 (58.8)
Male	1480 (41.2)
**Smoking Status**	
Never	774 (21.7)
Former	2078 (58.1)
Current	723 (20.2)
Unknown	16
**Histology**	
Nonsquamous	3181 (88.6)
Squamous	410 (11.4)
**Stage at NGS**	
I	862 (24.0)
II	280 (7.8)
III	522 (14.5)
IV	1927 (53.7)
**PD-L1 TPS**	
<1%	575 (34.3)
1-49%	607 (36.1)
≥50%	498 (29.6)
Not assessed	1911
**Tumor purity (%), median (range)**	40 (20-100)
**Genotype**	
No known driver	1242 (34.6)
KRAS	1141 (31.8)
EGFR	666 (18.5)
MET	170 (4.7)
BRAF	154 (4.3)
ALK	84 (2.3)
HER2	70 (2.0)
RET	38 (1.1)
ROS1	26 (0.7)

PD-L1, programmed death ligand 1
TPS, tumor proportion score
NGS, next generation sequencing

© 2022 Ricciuti B et al. *JAMA Oncology.*

eTable 2. Characteristics of Patients With NSCLC Treated With Immune Checkpoint Inhibitors at Memorial Sloan Kettering Cancer Center (MSKCC), Dana-Farber Cancer Institute (DFCI), and Stand Up To Cancer Foundation (SU2C)/Mark Foundation Dataset.

Clinical Characteristic	MSKCC N = 672 (%)	DFCI N = 714 (%)	SU2C N = 166 (%)
**Age, median (range)**	67 (22-92)	66 (25-92)	63.5 (39-86)
**Sex**			
Male	317 (47.2)	325 (45.5)	80 (48.2)
Female	355 (52.8)	389 (54.5)	86 (51.8)
**Smoking status**			
Current/Former	569 (84.7)	605 (84.7)	148 (89.2)
Never	103 (15.3)	109 (15.3)	18 (10.8)
**Histology**			
Non-squamous	586 (87.2)	629 (88.1)	132 (79.5)
Squamous	86 (12.8)	85 (11.9)	34 (20.5)
**Oncogenic driver mutation**			
KRAS	234 (34.8)	249 (34.9)	36 (21.7)
EGFR	67 (10.0)	71 (9.9)	5 (3.0)
Other	48 (7.1)	85 (11.9)	6 (3.6)
None identified	323 (48.1)	309 (43.3)	119 (71.7)
**ECOG performance status**			
0-1	622 (92.6)	563 (79.6)	0 (0.0%)
≥2	50 (7.4)	144 (20.4)	0 (0.0%)
Not available		7	166
**Line of therapy**			
1st	216 (32.1)	244 (34.2)	71 (42.8)
≥2nd	456 (67.9)	470 (65.8)	95 (57.2)
**PD-L1 expression**			
<1%	169 (40.9)	87 (17.0)	25 (26.0)
1-49%	78 (18.9)	171 (33.4)	38 (39.6)
≥50%	166 (40.2)	254 (49.6)	33 (34.4)
Not assessed	259	202	70

ECOG, Eastern Cooperative Oncology Group
NSCLC NOS, non-small cell lung cancer not otherwise specified
PD-L1, programmed death ligand 1
TMB, tumor mutational burden

© 2022 Ricciuti B et al. *JAMA Oncology.*

eTable 3. Tumor Mutational Burden (TMB) Values (in Mutations per Megabase, mut/Mb) at the Memorial Sloan Kettering Cancer Center (MSKCC), Dana-Farber Cancer Institute (DFCI), and Stand up To Cancer/Mark Foundation (SU2C) Cohorts Which Correspond With the Harmonized TMB Z-Score of 1.16.

	MSKCC (NGS)	DFCI (NGS)	SU2C (WES)
TMB (mut/Mb)	19.0	19.3	16.0
Percentile within cohort	89th	90th	88th

NGS, next-generation sequencing; WES, whole-exome sequencing

© 2022 Ricciuti B et al. *JAMA Oncology.*

eTable 4. Adjusted Odds Ratio for Response and Adjusted Hazard Ratio for Progression-Free and Overall Survival to PD-(L)1 Inhibition in the MSKCC Cohort After Multiple Imputation to Account for PD-L1 Missingness.

**Objective response**		
Variable	Odds Ratio [95% CI]	P value
Age	0.99 [0.97-1.01]	0.54
Sex (male vs female)	0.82 [0.53-1.24]	0.35
Smoking history (ever vs never)	2.10 [1.04-4.21]	**0.036**
Histology (squamous vs non-squamous)	1.27 [0.70-2.31]	0.42
ECOG PS (0-1 vs ≥2)	1.55 [0.61-3.94]	0.35
Line of ICI (1st vs ≥2nd)	0.71 [0.45-1.12]	0.14
PD-L1 TPS (≥50% vs <1%)	4.01 [2.33-6.89]	**<0.0001**
PD-L1 TPS (1-49% vs <1%)	1.95 [1.01-3.79]	**0.049**
TMB (high vs low)	3.08 [1.74-5.43]	**0.0001**
**Progression-free survival**		
Variable	Hazard Ratio [95% CI]	P value
Age	1.00 [0.99-1.01]	0.45
Sex (male vs female)	1.11 [0.94-1.31]	0.21
Smoking history (ever vs never)	0.69 [0.55-0.87]	**0.001**
Histology (squamous vs non-squamous)	1.04 [0.81-1.33]	0.73
ECOG PS (0-1 vs ≥2)	0.88 [0.65-1.19]	0.41
Line of ICI (1st vs ≥2nd)	1.24 [1.02-1.50]	**0.03**
PD-L1 TPS (≥50% vs <1%)	0.69 [0.56-0.84]	**<0.001**
PD-L1 TPS (1-49% vs <1%)	0.92 [0.69-1.21]	0.57
TMB (high vs low)	0.40 [0.29-0.55]	**0.0001**
**Overall survival**		
Variable	Hazard Ratio [95% CI]	P value
Age	1.01 [1.00-1.02]	**0.005**
Sex (male vs female)	1.10 [0.91-1.33]	0.29
Smoking history (ever vs never)	0.94 [0.73-1.20]	0.63
Histology (squamous vs non-squamous)	1.23 [0.95-1.60]	0.11
ECOG PS (0-1 vs ≥2)	0.73 [0.53-1.00]	0.05
Line of ICI (1st vs ≥2nd)	1.31 [1.06-1.62]	**0.01**
PD-L1 TPS (≥50% vs <1%	0.69 [0.54-0.87]	**0.002**
PD-L1 TPS (1-49% vs <1%)	0.88 [0.67-1.17]	0.41
TMB (high vs low)	0.51 [0.35-0.71]	**0.0001**

© 2022 Ricciuti B et al. *JAMA Oncology.*

eTable 5. Adjusted Odds Ratio for Response and Adjusted Hazard Ratio for Progression-Free and Overall Survival to PD-(L)1 Inhibition in the DFCI Cohort After Multiple Imputation to Account for PD-L1 Missingness.

**Objective response**		
Variable	Odds Ratio [95% CI]	P value
Age	1.01 [0.99-1.03]	0.16
Sex (male vs female)	0.72 [0.50-1.06]	0.09
Smoking history (ever vs never)	2.09 [1.11-3.94]	**0.02**
Histology (squamous vs non-squamous)	1.39 [0.79-2.44]	0.24
ECOG PS (0-1 vs ≥2)	3.07 [1.71-5.51]	**<0.001**
Line of ICI (1st vs ≥2nd)	0.84 [0.54-1.29]	0.43
PD-L1 TPS (≥50% vs <1%)	1.97 [1.03-3.76]	**0.04**
PD-L1 TPS (1-49% vs <1%)	0.99 [0.51-1.95]	0.99
TMB (high vs low)	3.20 [1.85-5.52]	**<0.0001**
**Progression-free survival**		
Variable	Hazard Ratio [95% CI]	P value
Age	0.99 [0.98-1.00]	0.07
Sex (male vs female)	1.22 [1.04-1.44]	**0.01**
Smoking history (ever vs never)	0.74 [0.59-0.92]	**0.009**
Histology (squamous vs non-squamous)	0.93 [0.73-1.19]	0.59
ECOG PS (0-1 vs ≥2)	0.51 [0.42-0.63]	**<0.0001**
Line of ICI (1st vs ≥2nd)	1.14 [0.93-1.39]	0.20
PD-L1 TPS (≥50% vs <1%)	0.63 [0.44-0.92]	**0.036**
PD-L1 TPS (1-49% vs <1%)	0.91 [0.69-1.20]	0.52
TMB (high vs low)	0.48 [0.36-0.65]	**<0.0001**
**Overall survival**		
Variable	Hazard Ratio [95% CI]	P value
Age	1.00 [0.99-1.01]	0.80
Sex (male vs female)	1.29 [1.06-1.51]	**0.01**
Smoking history (ever vs never)	0.87 [0.69-1.11]	0.29
Histology (squamous vs non-squamous)	1.01 [0.77-1.32]	0.92
ECOG PS (0-1 vs ≥2)	0.37 [0.29-0.45]	**<0.0001**
Line of ICI (1st vs ≥2nd)	1.37 [1.08-1.73]	**0.01**
PD-L1 TPS (≥50% vs <1%)	0.73 [0.48-1.12]	0.19
PD-L1 TPS (1-49% vs <1%)	1.04 [0.76-1.43]	0.78
TMB (high vs low)	0.51 [0.37-0.72]	**0.0001**

© 2022 Ricciuti B et al. *JAMA Oncology.*

eTable 6. Adjusted Odds Ratios for Response and Adjusted Hazard Ratio for Progression-Free and Overall Survival to PD-(L)1 Inhibition in the SU2C/Mark Foundation Cohort After Multiple Imputation to Account for PD-L1 Missingness.

**Objective response**		
Variable	Odds Ratio [95% CI]	P value
Age	1.02 [0.98-1.06]	0.28
Sex (male vs female)	0.78 [0.37-1.63]	0.51
Smoking history (ever vs never)	1.13 [0.34-3.72]	0.84
Histology (squamous vs non-squamous)	2.01 [0.77-5.26]	0.15
Line of ICI (1st vs ≥2nd)	0.48 [0.23-1.01]	0.06
PD-L1 TPS (≥50% vs <1%)	2.94 [0.94-9.18]	0.07
PD-L1 TPS (1-49% vs <1%)	1.34 [0.49-3.64]	0.56
TMB (high vs low)	25.8 [5.21-128.02]	**0.0001**
**Progression-free survival**		
Variable	Hazard Ratio [95% CI]	P value
Age	0.99 [0.97-1.01]	0.48
Sex (male vs female)	1.22 [0.83-1.78]	0.29
Smoking history (never vs ever)	1.20 [0.67-2.15]	0.54
Histology (squamous vs non-squamous)	0.99 [0.60-1.63]	0.97
Line of ICI (1st vs ≥2nd)	1.47 [0.99-2.19]	0.06
PD-L1 TPS (≥50% vs <1%)	0.61 [0.29-1.28]	0.22
PD-L1 TPS (1-49% vs <1%)	0.74 [0.44-1.26]	0.28
TMB (high vs low)	0.15 [0.06-0.35]	**<0.0001**
**Overall survival**		
Variable	Hazard Ratio [95% CI]	P value
Age	0.99 [0.97-1.02]	0.79
Sex (male vs female)	1.13 [0.72-1.76]	0.59
Smoking history (never vs ever)	1.92 [0.93-3.97]	0.08
Histology (squamous vs non-squamous)	0.86 [0.48-1.56]	0.63
Line of ICI (1st vs ≥2nd)	1.72 [1.06-2.78]	**0.03**
PD-L1 TPS (≥50% vs <1%)	0.56 [0.28-1.10]	0.11
PD-L1 TPS (1-49% vs <1%)	0.71 [0.40-1.23]	0.23
TMB (high vs low)	0.13 [0.04-0.43]	**0.001**

© 2022 Ricciuti B et al. *JAMA Oncology.*

eTable 7. Impact of TMB High Versus Low on Objective Response, Progression-Free, and Overall Survival in a Meta-analysis of the MSKCC and DFCI Cohorts. SU2C cohort was excluded as ECOG PS was not available for this cohort.

**Objective response (TMB high versus TMB low)**		
Model	Adjusted odds Ratio [95% CI]	P value
Complete dataset	2.90 [1.78-4.70]	<0.0001
Inverse probability weighting	2.93 [1.97-4.37]	<0.0001
Multiple imputation	3.14 [2.12-4.66]	<0.0001
**Progression-free survival (TMB high versus TMB low)**		
Model	Adjusted Hazard Ratio [95% CI]	P value
Complete dataset	0.47 [0.36-0.61]	<0.0001
Inverse probability weighting	0.47 [0.35-0.62]	<0.0001
Multiple imputation	0.44 [0.36-0.55]	<0.0001
**Overall survival (TMB high versus TMB low)**		
Model	Adjusted Hazard Ratio [95% CI]	P value
Complete dataset	0.59 [0.44-0.79]	0.0005
Inverse probability weighting	0.59 [0.43-0.82]	0.0014
Multiple imputation	0.51 [0.40-0.65]	<0.0001

© 2022 Ricciuti B et al. *JAMA Oncology.*

eReferences

1. Garcia, E. P. *et al.* Validation of OncoPanel: A Targeted Next-Generation Sequencing Assay for the Detection of Somatic Variants in Cancer. *Arch. Pathol. Lab. Med.* **141**, 751–758 (2017).
2. Cheng, D. T. *et al.* Memorial sloan kettering-integrated mutation profiling of actionable cancer targets (MSK-IMPACT): A hybridization capture-based next-generation sequencing clinical assay for solid tumor molecular oncology. *J. Mol. Diagnostics* (2015) doi:10.1016/j.jmoldx.2014.12.006.
3. Vokes, N. I. *et al.* Harmonization of Tumor Mutational Burden Quantification and Association With Response to Immune Checkpoint Blockade in Non–Small-Cell Lung Cancer. *JCO Precis. Oncol.* (2019) doi:10.1200/po.19.00171.
4. Aran, D., Hu, Z. & Butte, A. J. xCell: Digitally portraying the tissue cellular heterogeneity landscape. *Genome Biol.* (2017) doi:10.1186/s13059-017-1349-1.
5. Bogusz, A. M. *et al.* Quantitative Immunofluorescence Reveals the Signature of Active B-cell Receptor Signaling in Diffuse Large B-cell Lymphoma. *Clin. Cancer Res.* **18**, 6122–6135 (2012).
6. Hothorn, T., Hornik, K. & Zeileis, A. Unbiased recursive partitioning: A conditional inference framework. *J. Comput. Graph. Stat.* (2006) doi:10.1198/106186006X133933.
7. Kuhn, M. Building Predictive Models in R Using the caret Package. *J. Stat. Softw.* **28**, 1–26 (2008).

© 2022 Ricciuti B et al. *JAMA Oncology.*
